# Industrial-grade collaborative robots for motor rehabilitation after stroke and spinal cord injury: a systematic narrative review

**DOI:** 10.1186/s12938-025-01362-z

**Published:** 2025-04-30

**Authors:** Aisha Raji, Urvashy Gopaul, Jessica Babineau, Milos R. Popovic, Cesar Marquez-Chin

**Affiliations:** 1 KITE Research Institute, Toronto Rehabilitation Institute, University Health Network, 550 University Ave., Toronto, M5G 2A2 ON Canada; 2 Institute of Biomedical Engineering, University of Toronto, 164 College St., Toronto, M5S 3G9 ON Canada; 3 Library & Information Services, Toronto Rehabilitation Institute, University Health Network, 550 University Ave., Toronto, M5G 2A2 ON Canada

**Keywords:** Collaborative robot (cobot), Robotic rehabilitation, Stroke, Rehabilitation, Spinal cord injury, Upper extremity, Lower extremity

## Abstract

**Background:**

There is a growing interest in exploring industrial-grade collaborative robots (cobots) for rehabilitation. This review explores their application for motor rehabilitation of the upper and lower extremities after a stroke and spinal cord injury (SCI). The article highlights the inherent safety features of cobots, emphasizing their design advantages over custom-built or traditional rehabilitation robots in terms of potential safety and time efficiency.

**Methods:**

Database searches and reference list screening were conducted to identify studies relating to the use of cobots for upper and lower extremity rehabilitation among individuals with stroke and SCI. These articles were then reviewed and summarized.

**Results:**

Thirty-three studies were included in this review. The findings suggest that the use of cobots in motor rehabilitation is still in the early stages. Some of the cobots used were equipped with sensors to detect and respond to the movement of the extremities and minimize the risk of injury. This safety aspect is crucial for patients with motor impairments. Most training protocols implemented with the cobots engaged users in repetitive task-based exercises with an overall positive user experience. Thus far, these devices have been primarily evaluated in individuals with stroke and SCI that affect the lower extremities, with no study addressing upper extremity impairments. This initial focus serves as a preliminary step toward assessing their applicability for individuals with stroke and SCI.

**Conclusions:**

Cobots may have the capacity to transform therapy and support healthcare professionals in delivering more personalized and effective rehabilitation. However, there is limited evidence on their use to support upper and lower extremity rehabilitation among individuals with stroke and SCI. Further research and development are needed to refine these technologies and broaden their applications in rehabilitation settings to enhance functional recovery and overall quality of life for individuals with stroke and SCI.

**Supplementary Information:**

The online version contains supplementary material available at 10.1186/s12938-025-01362-z.

## Introduction

Stroke and spinal cord injury (SCI) are leading causes of disability worldwide. Over 101 million people have experienced a stroke, and more than 15 million people are currently living with SCI [1, 2]. These conditions often result in motor impairments, such as muscle weakness in the arms, hands, trunk, or legs, and loss of balance and coordination. These impairments can negatively impact an individual’s ability to perform activities of daily living (ADLs), such as walking, eating, and personal care, highlighting the need for effective rehabilitation [3–5].

Robotic rehabilitation has emerged as a promising intervention for addressing motor impairments in individuals with stroke and SCI. Optimal recovery often requires high doses of therapy; however, current rehabilitation programs frequently fall short of this goal due to heavy caseloads burdening therapists [6, 7]. Systematic reviews suggest that robotic rehabilitation effectively improves motor function, including gross motor skills, muscle strength, coordination, and motor control of the upper and lower extremities [6–9]. These devices can deliver intensive therapy characterized by varying levels of resistance, high repetition, and task-oriented rehabilitation. Often, these systems reduce the physical burden on therapists and are sometimes augmented with different techniques, such as virtual reality (VR), to engage patients in therapeutic activities [10, 11].

Robotic devices used in rehabilitation can be classified as exoskeletons or end-effector robots. End-effector robots make physical contact with patients at a single distal point, such as a handle or platform, to focus on specific movements [12]. End-effector robots target fine motor skills, muscle strengthening, balance and coordination. Examples include the MIT-MANUS/InMotion2 [13] and MIME [14] for upper extremity rehabilitation, and Gait Trainer [15] and HapticWalker [16] for gait rehabilitation. Exoskeletons are non-invasive wearable devices typically worn over multiple joints. They provide mechanical support and assistance by mimicking natural limb movements [17]. These robots support body weight, improve muscle strength, reduce spasticity, enhance motor control, and increase mobility [18–20]. Examples include the ARMin [21, 22] and Armeo Spring [23] for upper extremity rehabilitation, and Lokomat [24] and ReWalk [25] for lower extremity rehabilitation.

Rehabilitation robots can provide different modes of assistance, ranging from fully assisted motions to entirely patient-initiated movements, tailored according to the patient’s severity level and recovery stage. These modes include active, passive, active-assistive, and resistive exercises, all used to optimize rehabilitation outcomes [12]. In the active mode, patients voluntarily move their extremities. Conversely, in the passive mode, the robot performs the movement regardless of the patient’s capability or intention. The active-assistive mode involves patients actively performing movements, with the robot intervening if the patient’s movements are inadequate in terms of speed, timing, or force. Finally, in the resistive mode, the robot exerts force in the opposite direction of the patient’s movement, thereby increasing the difficulty of the exercise [26, 27].

Most rehabilitation robots are custom-made, but some upper extremity rehabilitation robots are adapted from industrial robot manipulators. For example, the Mitsubishi Pa10-7 robot platform uses a 7-degrees-of-freedom (DOFs) robot [28], the MIME uses a 6-DOF Puma-560 robot [14, 29], and the REHAROB uses the IRB 140 and IRB 1400 H robots from ABB Ltd. [30]. Amid the ongoing advancements in robotic rehabilitation, there has been notable interest in the potential of collaborative robots (cobots) for upper and lower extremity rehabilitation [31, 32].

### Cobots origin and usage

**Fig. 1:**
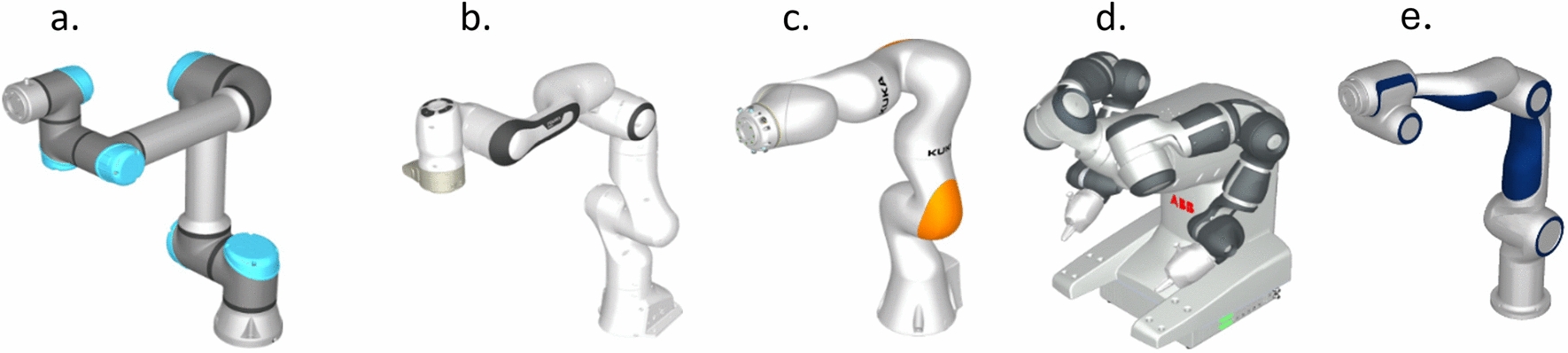
3D models of commercially available collaborative robots: (**a**) Universal Robot (UR5e), (**b**) Franka Panda Emika, (**c**) Kuka LBR, (**d**) ABB YuMi, and (**e**) Agile Robot (images courtesy of RoboDK) [37]

**Fig. 2:**
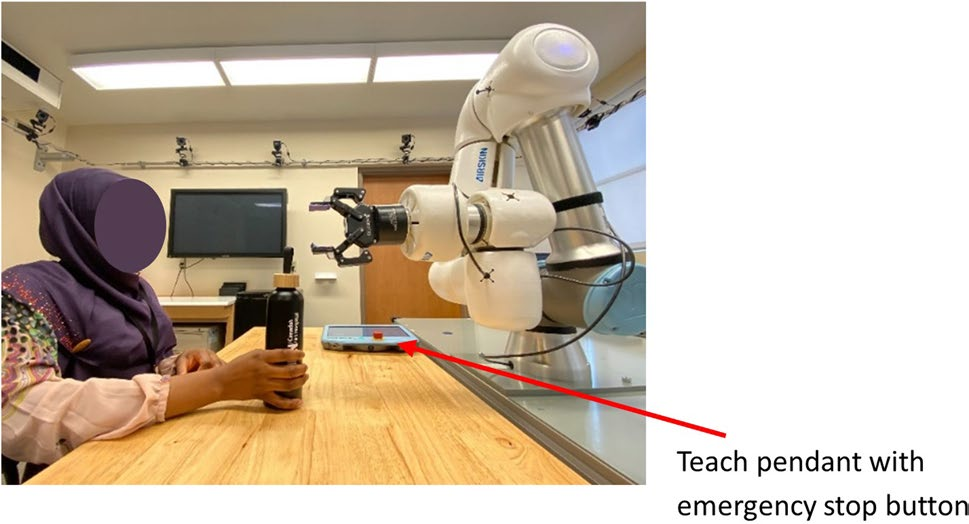
An illustration of the UR5e cobot

Cobots are commercially available industrial-grade robotic arms (Fig. 1) with built-in safety features that allow them to work safely alongside humans in the same workspace without physical barriers [33]. These devices originated in 1996 as passive systems, lacking actuators for autonomous movement and relied entirely on human input for guidance. Despite this, they were considered robotic due to their mechanical structures and precision mechanisms, which enhanced human capabilities in tasks requiring precision or strength [34, 35]. Today, cobots are fully actuated robotic systems that are sometimes indistinguishable from conventional industrial robots. Like industrial robots, cobots have essential components such as a control unit, a teach pendant, and an emergency stop mechanism, as shown in (Fig. 2), which makes it possible to program the robot and deal with emergencies. However, cobots are distinct due to their advanced safety features, which include low operational velocities, lightweight design, and force-feedback sensors, which allow for safe human–robot interaction. These features, combined with their ease of programming and flexibility, make cobots highly adaptable for various applications, such as assembly line customization in manufacturing and pick-and-place operations in logistics and warehousing [36].

### Cobots in rehabilitation

As cobots evolve, their applications are expanding into healthcare, particularly rehabilitation. Their adaptability and precision make them suitable for assisting in physical therapy and rehabilitation exercises, offering consistent and repeatable movements tailored to individual patient needs. The safety features and ease of programming that make cobots attractive in industrial settings also benefit rehabilitation potentially improving patient outcomes and reducing therapist workload.

For use in rehabilitation, the cobots’ last joint, known as the tool flange, serves as the mounting point for various tools and attachments, such as grippers or custom-made handles for ankle or arm supports. This flexibility allows cobots to perform specific rehabilitation tasks, ranging from fine motor skill activities to gait training [31, 32, 38]. Cobots meet rigorous safety standards, providing an advantage over custom-built robotic rehabilitation devices, which require separate safety modules and additional development time and cost [13, 21].

Building on these advantages, the overall aim of this systematic narrative review of the literature is to explore the use of cobots in motor rehabilitation of the upper and lower extremities after a stroke or SCI. The objectives of this review are

1. To explore the characteristics of cobots and how they are adapted to deliver motor rehabilitation of the upper and lower extremities after stroke or SCI.
2. To review the training protocols used by cobots to deliver motor rehabilitation to the upper and lower extremities after stroke or SCI.
3. To determine the outcomes of motor rehabilitation of the upper and lower extremities after stroke or SCI, delivered by cobots.

## Methods

### Search strategy

An extensive search of electronic databases was conducted by JB seeking to identify suitable publications. The search was first developed in MEDLINE ALL (Ovid) and subsequently translated to the following databases: Embase (Ovid), Cochrane Central Register of Controlled Trials (CENTRAL, Ovid), IEEE Xplore, ACM Digital Library, Compendex (Engineering Village), INSPEC (Engineering Village), Scopus, Dissertations and Theses (Proquest), and the Web of Science Core Collection. The search strategy consists of multiple concepts, including cobots, rehabilitation, stroke, SCI, Upper Extremities and Lower Extremities, combined with Boolean operators and using keywords and database-controlled vocabulary (e.g. MeSH, Emtree). See Appendix A for further details on the strategy, including synonyms for each concept. Searches were limited to English language publications when possible. Searches were originally conducted in February 2023 and updated in November 2024. In addition to comprehensive database searching, the reference lists of the papers included were reviewed to identify additional papers.

### Eligibility criteria

Studies were selected for inclusion based on the following criteria: (a) publications in peer-reviewed journals; (b) research focusing on upper or lower extremity rehabilitation following stroke or SCI, including research with healthy participants to determine how a cobot was intended to be used with people with stroke or SCI; and (c) access to abstracts and full papers. We excluded articles that focused on conditions other than stroke or SCI, involved using cobots for anything other than rehabilitation, participants were under the age of 18, were conducted in any language other than English, or had previous reviews or meta-analyses.

### Analysis

The narrative review examined the name of authors and date of publication, the aim of the study, the specific cobot used, participants population, including the targeted extremities, modifications made to the cobot, methods used, and key intervention outcomes.

## Results

### Search and selection

**Fig. 3:**
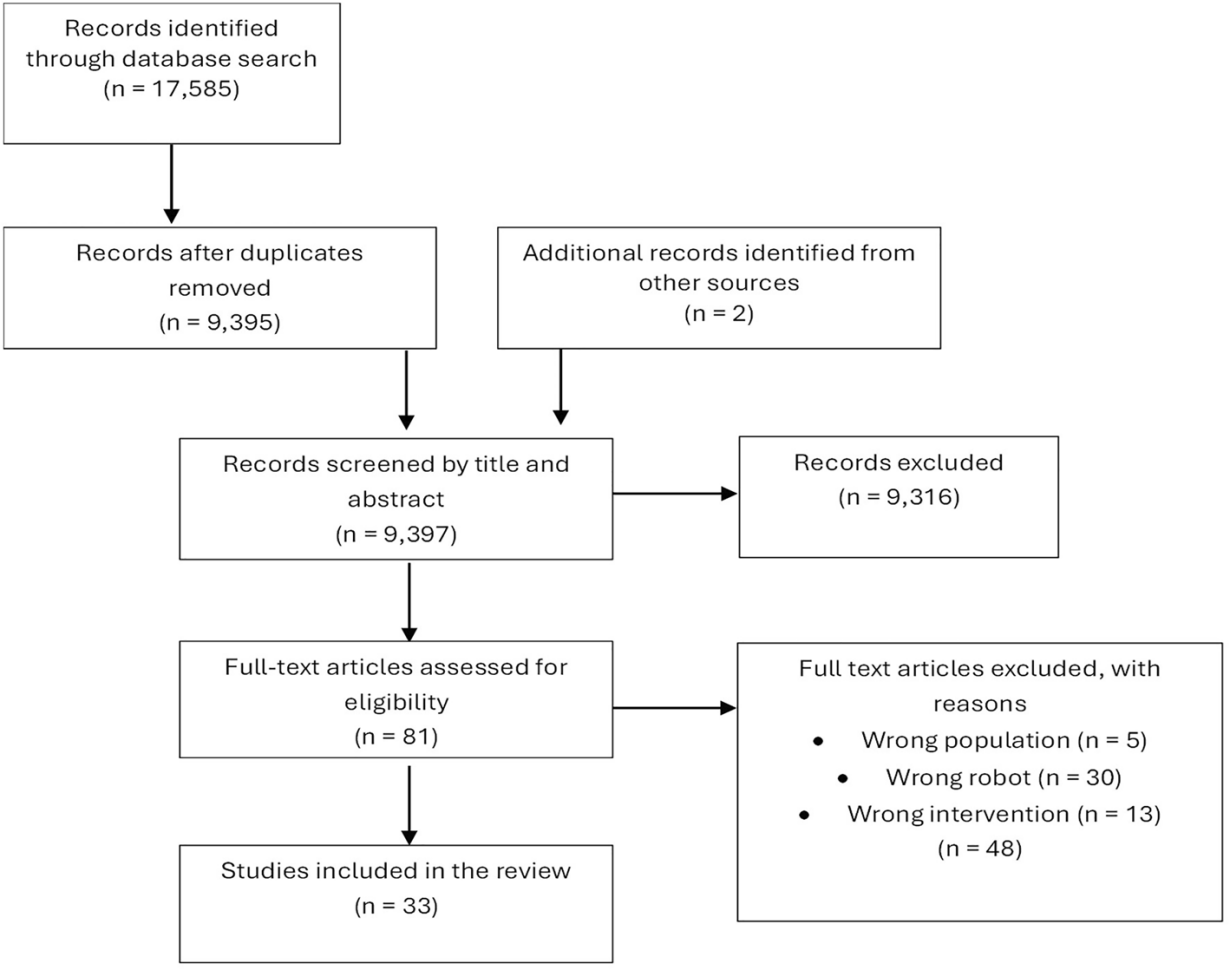
PRISMA flow diagram of the results from the database searches

We identified 17,585 studies in our search, 7,171 of which were duplicates. Two authors, AR and UG, independently screened the deduplicated search results and evaluated the titles and abstracts. AR retrieved the full papers of potentially eligible references, and then both AR and UG assessed the eligibility of these articles. Figure 3 shows the PRISMA flow diagram of the database search and screening results.

After screening titles and abstracts, we excluded 9,316 articles and examined the full text for the remaining studies. Two additional publications were found through manual searches of the reference list of the included studies.

### Characteristics of the included articles

**Fig. 4:**
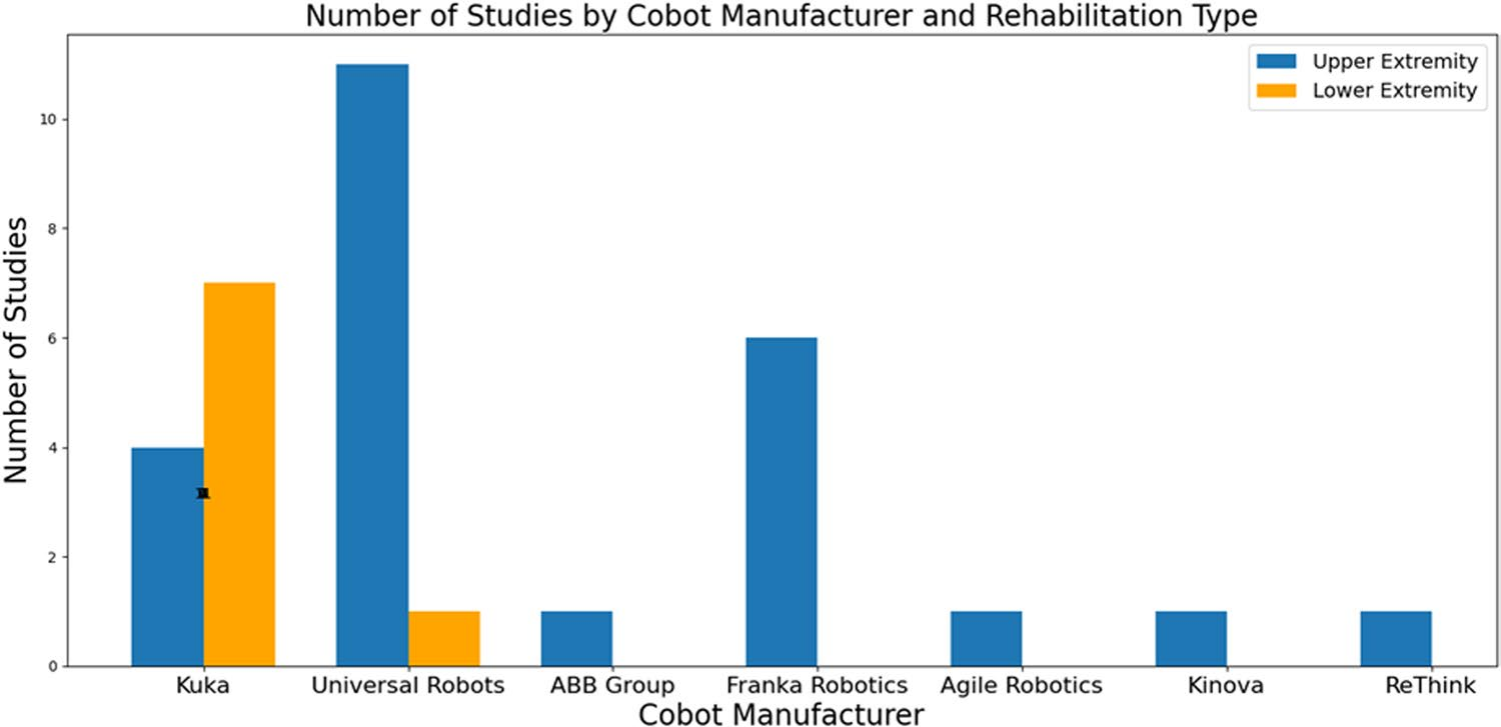
Studies by cobot manufacturer and rehabilitation type

The final result included 33 studies published from 2013 to 2024. The cobots identified in these studies include the Franka Emika Panda (Franka Robotics$\circledR$, Germany), ABB YuMi (ABB$\circledR$, Switzerland), Kuka LBR (KUKA$\circledR$, Germany), Sawyer (The HAHN Group$\circledR$, Germany), Agile robotic (Agile Robots$\circledR$, Germany), Kinova (Kinova$\circledR$, Canada), and Universal Robots (UR) series (Universal Robots$\circledR$, Denmark). As shown in Fig. 4, the distribution of studies by cobot manufacturer and rehabilitation type highlights that the Universal Robots (UR) series was prominently featured, with 12 articles reported [31, 39–49]. Kuka LBR cobots were also predominatly featured in 13 articles [32, 38, 50–60], four articles used the Franka Emika Panda [61–64], while Sawyer [65], Agile robot [66], Kinova [67], and ABB YuMi [68] were used in fewer articles.

Twenty-five articles used the cobots to implement various robotic rehabilitation exercises for the upper extremity [31, 39–47, 49–55, 61–68], while eight focused on the the lower extremity [32, 38, 48, 56–60] rehabilitation. Geographically, 17 of the studies were conducted in Europe [31, 32, 38–40, 46, 48–51, 53, 56–59, 63, 68], 10 in Asia [47, 52, 54, 55, 60–62, 64–66], 1 in South America [41], 3 in North America [42, 45, 67], and 2 in Oceania [43, 44].

### Description of the cobots found in the included studies

The characteristics of the cobots found in the studies included in this review are displayed in Table 1. The key specifications of these cobots are as follows:

- *Weight:* total weight of the robot, which affects its stability and portability.
- *Payload:* maximum load that the robot can handle safely.
- *Pose repeatability:* The robot’s precision to return to a specific position.
- *Robot reach:* Maximum working range from the base to the end of the arm or tool.

Most studies focusing on lower extremity rehabilitation have utilized ROBERT$\circledR$, a portable robotic rehabilitation device based on the KUKA cobot platform developed by Life Science Robotics for both upper and lower extremity therapy [69]. Equipped with seven degrees of freedom, the system delivers active resistive and assistive mobilization, thereby facilitating early patient movement and promoting neuroplasticity. ROBERT$\circledR$ is classified as a Class IIa medical device and is duly registered with both the FDA and MHRA.

**Table 1:** Characteristics of the cobots found in the studies

Characteristics	Universal Robots (UR) Series	KUKA	Franka Emika Panda	ABB IRB 14000 YuMi	Sawyer	Agile	Kinova
Degrees of Freedom	6	7	7	7 per arm	7	7	6
Payload Capacity (kg)	UR3: 3 kg, UR5: 5kg, UR10: 10kg	LWR4+: 7kg, LBR iiwa 7: 7 kg	3 kg	0.5 kg per arm	4 kg	7 kg	2.6kg
Robot Weight	UR3: 11 kg, UR5:18.4 kg, UR10: 28.9 kg	LWR 4+: 16 kg, LBR iiwa 7: 23.9 kg	18 kg	38 kg (entire robot)	19 kg	26 kg	4.4 kg
Robot Reach	UR3: 500 mm, UR5: 850 mm, UR10: 1300 mm	LWR 4+: 790 mm, LBR iiwa 7: 800 mm	855 mm	559 mm per arm	1260 mm	923 mm	985 mm
Pose Repeatability	± 0.1 mm	LWR 4+: ±0.05 mm, LBR iiwa 7: ± 0.1 mm	± 0.1 mm	0.02 mm	± 0.1 mm	± 0.05 mm	±0.03 mm
Controller	Control box Via the Teach Pendant, Programming Iinterface	KUKA Sunrise Cabinet via The SmartPAD, Programming Interface	Software API	IRC5 Controller Via the FlexPendant Programming Interface	Intera	Agile Control System	Kortex
Joint Working Range	±360°	±120° - ±170°	±166° - ±215°	$\pm$ $\pm$ $\pm$88° - ±290°	341° - 540°	±179°	±135°
Programming languages	URScript, Python, ROS, C++, MATLAB, Teaching Through Demonstration	Java, C++, Python, MATLAB, Teaching Through Demonstration	C++, Python, MATLAB	RAPID, Python, ROS, MATLAB, Teaching Through Demonstration	Intera Studio, C++, Python	C ++, Python, MATLAB	Kortex API, C++, MATLAB
Safety Feature	Force Sensing, Emergency stop	Force detection, emergency stop	Force sensing, emergency stop	Dual arm with Collision detection, emergency stop	Force Sensing, ergency Stop	Force Sensing, Emergency stop	Force sensing, emergency Stop
Studies Found	[31, 39–49]	[32, 38, 50–60]	[61–64]	[68]	[65]	[66]	[67]

API = Application Programming Interface

**Fig. 5:**
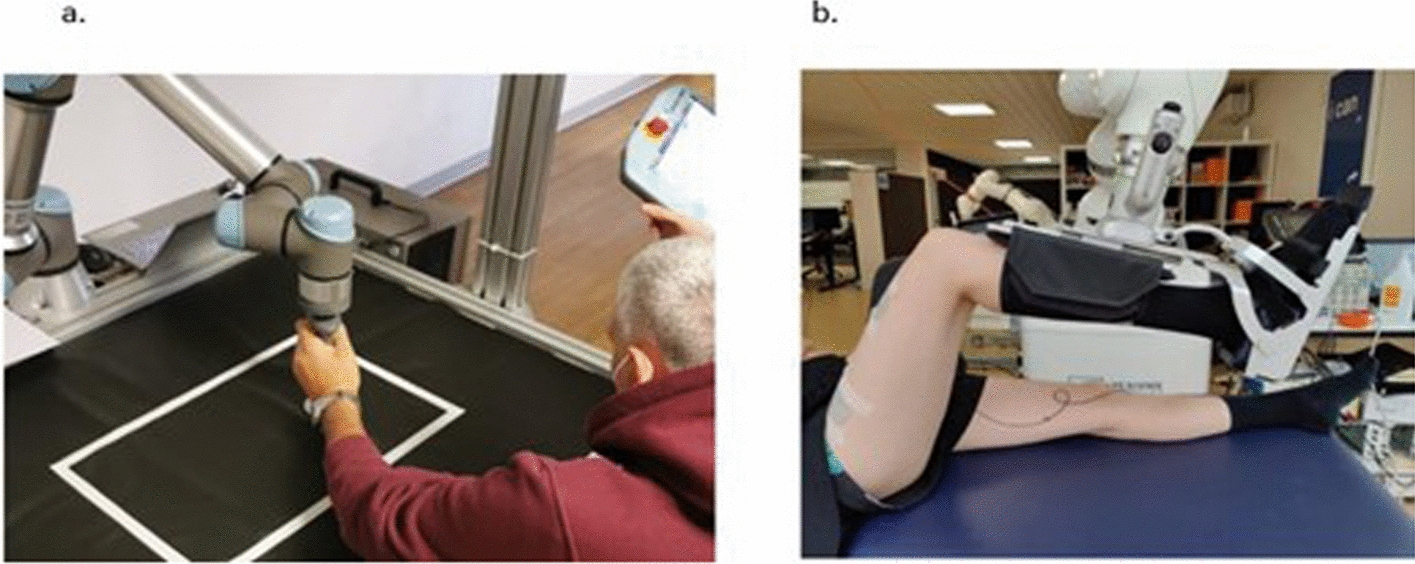
Examples of how cobots are used for (**a**) upper extremity, ©2023 by Chiriatti et al. Licensee MDPI, Basel, Switzerland [46] and (**b**) lower extremity rehabilitation, ©2024 by Leerskov et al. Published by Elsevier Ltd. [57]. These images are licensed under the CC BY license (http://creativecommons.org/licenses/by/4.0/)

Figure 5 shows how cobots are used in motor rehabilitation while Table 2 summarizes the devices used, modifications, and findings of the studies included in this review.

**Table 2:** Summary of the articles included in the review

Authors, year of publication, and Reference	Cobot used	Cobot Adaptation	Cobot Aim	Participant population	Findings
Kyrkjebø et al., 2018 [31]	UR5	Force sensor, virtual boundary	Feasibility of cobot for rehabilitation	Simulations	The UR5 cobot is capable of functioning in both assistive and resistive modes. With the implementation of appropriate safety boundaries and control strategies, the UR5 may support rehabilitation training.
Gherman et al., 2019 [68]	ABB IRB 14000 YuMi	3D-printed custom gravity- compensation support	Application of cobot for upper limb rehabilitation	N = not specified	With an appropriate arm support and predefined trajectory, the ABB IRB 14000 YuMi is feasible fo targeted rehabilitation exercises for the upper extremity.
Fortini et al., 2019 [63]	Franka Emika Panda	Eye-tracker, sEMG sensor	To develop a robotic assistive-reaching system that uses eye- tracking technology to guide users in reaching various targets	N = 10 healthy participants	The system enables users to intuitively and accurately perform reaching tasks without prior training or physical skills.
Majidirad et al., 2018 [42]	UR5	Force sensor, gripper, customized knob, gripper, sEMG	To assess the effectiveness of cobot intervention in a task- based upper extremity rehabilitation exercise	N = 5 healthy participants	There is a correlation between muscle activation patterns in the upper extremity based on force data from the robot and the signal recorded by the sEMG sensors.
Sørensen et al., 2014 [40]	UR5	Force sensor and customized handle	To mimic real-world rehabilitation exercises	Simulations	The UR5 cobot can replicate the functionality of different training devices like ‘curl’ and ‘rope.’
Majidirad et al., 2020 [45]	UR5	Ergonomic knob, Force sensor, gripper, sEMG sensor, IMU	To develop a novel and controlled intervention procedure for upper extremity task-based rehabilitation	N = 5 healthy participants	A strong correlation exists between force exertion and muscle activities in the upper extremity during task- based exercises.
Kato et al., 2020 [62]	Franka Emika Panda	Custom elbow support, sEMG sensor, virtual reality display with haptic feedback	To investigate how target difficulty and haptic feedback influence muscle activities of the upper extremity during assisted reaching exercises	N = 1 healthy participant	Different distances to the target, target size, and haptic feedback influenced the intensity of muscle activities during reaching tasks.
Scotto di Luzio et al., 2018 [51]	Kuka LWR 4+	Motorized arm- weight support, ergonomic arm brace, sEMG sensor, M-IMU	To develop a novel 3D bio-cooperative robotic strategy for upper extremity rehabilitation that includes the patient within the control loop	N = 10 healthy participants	The system is capable of reducing muscle fatigue by providing assistance based on user’s biomechanical and physiological measurements.
Nielsen et al., 2017 [39]	UR5	Force sensor, wrist support, customized handle	To develop a novel approach for training upper extremities after a stroke using an industrial robotic arm and dynamic movement primitives (DMPs) with force feedback	Simulations	The novel approach is feasible for personalized and adaptive training of the upper extremities for individuals with different levels of impairments.
Papaleo et al., 2013 [50]	Kuka LWR 4+	3D printed custom wrist support, M-IMU sensors	To develop and validate a patient-tailored adaptive robotic system for upper extremity rehabilitation	N = 2 healthy participants	The adaptive system can safely adjust to individual needs, enhance user engagement through performance indicators, and may improve therapy outcomes through analysis of the user’s biomechanical data.
Zhang et al., 2021 [61]	Franka Emika Panda	3D-printed customized handle	To propose an online reference path generation method for upper extremity rehabilitation robots that considers the initial motions of the participants	N = 10 healthy participants	This adaptive model could provide assistance as needed and effectively generate accurate reference paths for point-to-point tasks based on the initial motions of the participants.
de Azevedo Fernandes et al., 2020 [41]	UR3	3D printed tool, force sensor	To demonstrate the effectiveness of upper extremity rehabilitationusing an intelligentsystem that learns andadapts its behaviourbased on the patient’s performance during therapy sessions.	N = 1 healthy participant	The system is able to learn and dynamically adapt to varying user forces.
Miao et al., 2018 [44]	UR5 and UR10	Force sensors and customized handlebars	To propose a three- stage trajectory generation method for robot-assisted bilateral upper limb training with subject-specific adaptation	N = 7 healthy participants	The proposed method effectively provided safe and individualized bilateral upper extremity training exercises.
Sheng et al., 2019 [43]	UR5 and UR10	Force sensors and customized handlebars	To develop and evaluate a bilateral upper extremity rehabilitation system based on modern industrial robots	N = 10 healthy participants	The cobots used are feasible in providing bilateral upper extremity rehabilitation exercises.
Becker et al., 2018 [53]	Kuka LBR iiwa 14	Customize hand brace, impedance control	To implement the Assist-as-Needed (AAN) principle for adaptive upper extremity therapy by adjusting support based on position, velocity, and force.	N = 10 healthy participants	The cobot provided movement quality and consistent comfort, while smoothness improved with increased effort.
Zhang, Guo, & Sun 2020 [54]	Kuka LBR iiwa 14	Custom handles, display screens, cameras, and a body structure module	To enhance upper extremity rehabilitation by providing adaptive assistance through a virtual stiffness gradient	N = 1 healthy participant	The system demonstrated good trajectory adherence and controlled interaction forces with AAN.
Lim et al., 2023 [47]	UR robot	Gripper, custom hand brace	To facilitate in-home therapy for individuals with motor disabilities, targeting both gross and fine motor skills training	N = 5 healthy participants	The system showed significant muscle activation during robot-guided exercises compared to therapist- assisted training. Participants reported high satisfaction regarding training efficacy and system safety.
Behidj et al., 2023 [67]	Kinova MICO Gen 2	Custom- designed hand support	To develop and implement a position-based impedance control algorithm for a 6-DOF upper limb rehabilitation robot, thereby eliminating the necessity for external force sensors	N = Not specified	The system demonstrated the capability to adjust robot compliance and support dynamically, enhancing access to upper extremity rehabilitation and potentially reducing caregiver demands.
Pezeshki et al., 2023 [64]	Franka Emika Panda	Customized hand brace, display screen	To encourage patient participation and enhance the effectiveness of training sessions by minimizing robot intervention while following a predefined path	N = 4 healthy participants	The AAN strategy enhances user engagement, supports active and passive modes, and provides resistance-based challenges.
Liu et al., 2023 [66]	Agile cobot	Hand straps, custom handle	To enable stroke patients achieve accurate, real-time, and stable rehabilitation outcomes through a human–machine interaction system based on a 7-DOF cobot arm	N = Not specified	The system effectively identified force magnitude and direction and halted the arm’s movement when force exceeded a predefined threshold. This demonstrates the potential for enhancing user engagement and trajectory control in rehabilitation.
Rodrigues et al., 2023 [49]	UR cobot	Augmented reality, HoloLens headset, Unity game engine	To enhance upper extremity rehabilitation through gamification and interactive experiences	N = 31 healthy participants	The system was user-friendly, significantly enhanced participant motivation, and received high therapist satisfaction due to its customizable features.
Shi & Luo 2024 [65]	Sawyer cobot	A haptic device, sEMG sensor	To create a teleoperation framework that assists patients with upper limb hemiplegia controlling a slave robotic arm to perform specific passive and active rehabilitation training	N =Not specified	The system achieved smooth trajectory reproduction without abrupt stops, effectively generated guiding virtual forces, and highlighted the potential benefits of cobot-assisted rehabilitation.
Chiriatti et al., 2023 [46]	UR5e	Specialized handle	To present a rehabilitation framework for upper limbs of neurological patients, utilizing a collaborative robot to assist users in performing a given three-dimensional trajectory	N = 1 healthy participant	Preliminary tests with healthy participants demonstrated the system’s intuitiveness, user-friendliness, and effective safety measures.
Zhang, Guo, & Sun 2020 [55]	Kuka LBR iiwa 14	Custom-made handle	To develop and validate an assist-as-needed (AAN) controller for robot-aided rehabilitation training of the upper extremity, which can adapt to different rehabilitation modes such as passive, assistive, active, and resistive training.	N = 1 healthy participant	The AAN controller effectively facilitated task completion, promoted active engagement, enhanced motion performance, and demonstrated potential for improving motor recovery in individuals with upper extremity impairments.
Ai et al., 2023 [52]	KUKA LBR iiwa R700	Custom handle, motion capture system	To develop and validate the Uncertainty Compensated High- Order Adaptive Iteration Learning Control (UCHAILC) for improving trajectory tracking in robot- assisted upper limb rehabilitation, particularly for stroke patients.	N = Not specified	The UCHAILC method enhanced tracking performance, enabling more accurate and personalized assistance. This approach may improve rehabilitation outcomes by offering task-specific, repetitive training aligned with individual patient needs.
Tucan et al. 2021 [32]	KUKA LBR iiwa	3D printed leg constraining and sole plates	To develop a robotic system that uses a cobot equipped with a specially designed device for ankle rehabilitation	N =1 healthy participant	The cobot used is feasible for delivering ankle rehabilitation exercises.
Peterson et al.,2020 [38]	ROBERT$\circledR$ (Kuka)	Leg brace, sEMG electrodes, FES electrode	To implement and evaluate a novel sEMG- triggered functional electrical stimulation (FES) hybrid robotic rehabilitation system for enhancing lower limb rehabilitation in stroke patients	N = 10 healthy participants	The system achieved high success in exercise repetitions and force generation, indicating that sEMG- triggered FES combined with robotic assistance may enhance rehabilitation and functional recovery.
Leerskov et al., 2022 [59]	ROBERT$\circledR$ (Kuka)	Leg brace, sEMG electrodes, FES electrode	To systematically investigate the extent of both potentiation and fatigue in velocity and interaction force during repetitive ROBERT$\circledR$-FES exercising	N = 8 healthy participants	The results demonstrated varied responses: 50% of participants showed potentiation (increased velocity and force), while 50% experienced fatigue, emphasizing the need for adaptive rehabilitation systems.
Leerskov et al., 2024 [58]	ROBERT$\circledR$ (Kuka)	FES electrodes, EMG electrodes, foot brace	To evaluate the technical performance and clinical feasibility of integrating the ROBERT$\circledR$ with FES within an AAN strategy for the rehabilitation of lower extremity function in stroke patients	N = 10 healthy participants and 2 individuals with stroke	The system achieved over 96% accuracy in behavior detection and assistance modulation, enhanced engagement, voluntary effort, and motor learning in post-stroke participants.
Rikhof et al., 2024 [57]	ROBERT$\circledR$ (Kuka)	FES electrodes, EMG electrodes, foot brace	To assess the feasibility of combining robotics and FES with an AAN approach to support actively-initiated leg movements in (sub-) acute stroke patients	N = 9 individuals with subacute stroke	Assistance was needed in 44% of ankle dorsiflexion and 5% of knee extension repetitions, with mild to moderate fatigue, demonstrating the feasibility of integrating robotics and FES for stroke leg rehabilitation.
Sórensen et al., 2024 [56]	ROBERT$\circledR$ (Kuka)	Leg brace	To determine the feasibility of conducting a large trial designed to determine whether the ROBERT$\circledR$ can be used to increase strength in the hip flexor muscles after SCI.	N = 4 individuals ith SCI	The study reported a 92% training adherence, no adverse events, and positive feedback, suggesting ROBERT$\circledR$ is acceptable and potentially effective for enhancing hip flexor strength.
Wolański et al., 2023 [48]	UR10	Leg brace, Noraxon My- oMotion system, IMU sesnors	To evaluate the feasibility of adapting the UR10e industrial robot to assist in the rehabilitation process, specifically focusing on its ability to perform Proprioceptive Neuromuscular Facilitation (PNF) movements and comparing its performance to that of a physiotherapist	N = Not specified	The cobot executed repetitive exercises, with movement accuracy dependent on trajectory programming, and demonstrated potential efficiency advantages in cobot- assisted rehabilitation.
Chan 2022 [60]	ROBERT$\circledR$ (Kuka)	Not specified	To evaluate the cost- effectiveness and clinical benefits of the ROBERT$\circledR$ robotic rehabilitation device for lower limb rehabilitation for stroke patients	N = Not specified	ROBERT$\circledR$ may reduce hospital stays and readmissions, incur only one-tenth the operating costs of outpatient sessions, and provides a cost- effective rehabilitation solution in clinical settings.

sEMG: surface electromyography, IMU = inertial measurement unit, M-IMU = magneto-inertial measurement units

### Upper extremity rehabilitation

#### Unilateral Upper Extremity Rehabilitation

##### Cobot Control with Force Sensor and Internal Safety Configurations

The use of the UR5 cobot for robotic rehabilitation was evaluated in a feasibility study [31]. To enhance safety, the cobot was fitted with an external force/torque sensor, and limits were set to stop the robot automatically if exceeded. Simulated ADL tasks such as reaching and drinking were used to evaluate the cobot’s safety and efficacy. The cobot operated in assistive and resistive modes. Although testing was not conducted with a patient population, the results suggested that the UR5 could effectively support stroke rehabilitation with proper safety and control strategies.

In a different study, a UR5 cobot was equipped with a 1-degree-of-freedom force sensor and a customized handle [40]. The modification aimed to simulate traditional rehabilitation devices like the ‘curl’ and ‘rope’ by programming the cobot to move the arm along a predefined path, mimicking real-world rehabilitation exercises with resistive training assistance. A qualitative comparison of the cobot’s performance against specific devices was conducted without human subjects. The findings highlighted the UR5’s ability to replicate the functionality of various training devices, demonstrating its potential use in rehabilitation settings.

Another study used the ABB IRB 14000 YuMi cobot, equipped with a custom arm support and handle, compensated for gravity and guided users along predefined trajectories [68]. It offered passive and active-assistive rehabilitation modes with adjustable force sensitivity for different patient needs. Testing with healthy participants showed the cobot’s feasibility for upper extremity rehabilitation, highlighting its precision and adaptability in delivering targeted exercises.

The UR robotic arm was designed to be used for in-home upper extremity therapy for individuals with motor disabilities [47]. Equipped with a custom forearm-mounted holder, a gripper, sEMG, force/torque sensors, RGB-depth cameras, and an emergency stop activated if the force exceeded 45N, the system used imitation learning to adapt therapist-recorded trajectories to the user’s capabilities. Five healthy participants performed passive and assistive fine and gross motor exercises in a remote adaptive setup, with exercise trajectories simulated in Gazebo and OpenSim for safety. sEMG analysis indicated significant muscle activation during robot-guided exercises compared to therapist-assisted training. Participants also reported high satisfaction with system safety and performance.

Another study explored impedance-based control in a Kinova MICO Gen 2 cobot for upper extremity rehabilitation [67]. The system, equipped with a custom-designed hand support and operating without external force sensors, dynamically adjusted compliance and support based on user performance and recovery stage while following predefined trajectories. One healthy participant performed passive shoulder extensions, resistive tasks, and ADL, with the cobot providing appropriate assistance in response to deviations. System performance, evaluated through response time, overshoot, and position error, demonstrated effective compliance adjustments. These findings suggest that this approach could improve rehabilitation accessibility for people with stroke and reduce caregiver burden.

A real-time movement intention recognition system was integrated with an Agile cobot for upper extremity rehabilitation [66]. The user’s hand was strapped to the robot, allowing the system to interpret the force magnitude and direction exerted by the palm to control robot movements. An algorithm analyzed the angle between the resultant force at the robot’s tool center point (TCP) and the tangent direction of the position point to determine movement intention. Experimental results demonstrated the system’s ability to detect force magnitude and direction, stopping the arm when forces exceeded a predefined threshold. This offers a promising solution to improve patient engagement and trajectory control in rehabilitation.

##### Cobot Control with Assist-As-Needed (AAN) Strategy

A study evaluated the KUKA LBR iiwa 14 cobot for implementing the AAN principle using impedance control [53]. The system adapts upper extremity therapy by adjusting support based on position, velocity, and force. Physiotherapists initially guided users in performing ADLs, which were later completed independently with robotic assistance. Ten healthy subjects performed wiping and hand-to-head movements under three adaptation strategies with varying personal effort levels (0%, 50%, and 100%). Although the movement quality, comfort, and trajectory were maintained, movement smoothness improved with increased personal effort. The system shows promise for stroke rehabilitation but requires further clinical validation.

Another study developed an AAN robotic rehabilitation system using a Franka Emika cobot to enhance patient engagement and training effectiveness [64]. The cobot, equipped with a customized hand brace, was used to monitor interaction forces and estimate user intention, a display screen for path trajectory, and an AAN control algorithm to dynamically adjust assistance. Four healthy participants performed passive, active trajectory-following, and resistive exercises. Results showed enhanced patient engagement with the system, which supports active, passive, and resistance-based modes.

An upper extremity rehabilitation system was developed using two KUKA LBR iiwa 14 cobots equipped with custom handles, display screens, cameras, and a body structure module [54]. The system, controlled by an AAN controller, activates the cobot based on the affected extremity and provides assistance only when deviations exceeded a defined virtual channel. One healthy subject performed a circular trajectory task under three conditions: no assistance, force-based, and AAN-based. Results show improved trajectory adherence and controlled interaction forces with AAN, though further clinical validation is necessary to confirm its efficacy for rehabilitation.

In a follow-up study, the authors developed and validated an AAN controller for the two KUKA LBR cobots, extending its capabilities to passive, active, assistive, and resistive upper extremity training modes [55]. A custom end-effector handle allowed users to attach their hand directly to the cobot, enabling movement within a predefined fault-region. Outside this region, the robot provided assistance tailored to individual abilities by adjusting stiffness and assistance parameters. A preliminary evaluation with one healthy participant involved performing circular trajectories displayed on a screen with varying assistance levels. The results demonstrated that the AAN controller effectively facilitated guided task completion, promoted active engagement, and improved motion performance, suggesting its potential to enhance motor recovery in individuals with upper extremity impairments.

##### Cobot Control with Electrophysiological Data

Several studies have explored cobots for surface electromyography (sEMG)-based therapy using sensors like inertial measurement units (IMUs) [42, 45, 62, 63]. A study used a UR5 cobot with a force sensor, gripper, and ergonomic knob to assist users during arm movements while recording muscle activity via sEMG [42]. Although this study involved only five healthy participants performing exercises with and without robotic assistance, the results showed a significant correlation between muscle activity and robotic force. Despite the small sample size, these findings are valuable as they suggest that cobots can effectively support motor rehabilitation by providing real-time feedback on muscle engagement and force application through sEMG, highlighting their potential for integration into personalized rehabilitation therapies.

In a different study, the authors showed that the UR5 cobot can enhance upper extremity muscle recruitment without introducing force or stiffness challenges to the patients [45]. Using sEMG and IMUs to monitor muscle fatigue, five healthy participants performed predefined circular arm movements in task-based exercises. The results showed a strong correlation between force exertion and muscle activity, suggesting that precise force adjustments by the cobot could significantly improve muscle engagement and the effectiveness of rehabilitation exercises.

Another study used the Franka Emika Panda cobot with a custom handle and eye-tracker to develop a gaze-based interface for unilateral upper extremity rehabilitation [63]. The system used sEMG signals and eye tracking to guide the robot’s movements, reducing the need for assistance. Ten healthy participants performed gaze-guided reaching tasks, highlighting the system’s ability to facilitate intuitive and accurate task performance, reduce physical effort, and improve repetitive task training during rehabilitation.

##### Cobot Control with Gamification and Virtual Reality

Some studies integrated cobots with virtual reality (VR), sEMG sensors, and IMUs [49, 51, 62]. The Franka Emika Panda cobot, paired with sEMG sensors, was used to study how target difficulty and haptic feedback affect muscle activation [62]. An orthosis and an electro-holding magnet were used to restrict wrist movement, and an elbow support was used to reduce arm fatigue. A healthy participant performed reaching tasks in a virtual environment with and without haptic feedback. The results showed that target difficulty and haptic feedback significantly influenced muscle activation, highlighting the importance of task complexity in rehabilitation.

Similarly, the Kuka LWR 4+ cobot was used to develop a 3D bio-cooperative robotic platform with a motorized arm-weight support [51]. The system used the cobot, EMG sensors, and magneto-inertial measurement units (M-IMUs) to track arm movements, evaluate fatigue, and adjust assistance in real-time. Ten healthy participants controlled a VR hand avatar for 2D and 3D exercises. The study showed that the platform significantly reduced muscular fatigue without affecting motor patterns, suggesting its potential for personalized therapy.

A study developed an augmented reality (AR) application for a UR cobot—real or simulated—to enhance upper extremity rehabilitation through gamification and interactive experiences [49]. Built using the Unity$\circledR$ game engine, the system integrated with the HoloLens headset to enable virtual object visualization, head movement tracking, and translation into the virtual environment. Therapists defined and customized therapeutic trajectories based on patient needs, with the robot as an assistive tool. A usability evaluation with 31 healthy participants assessed the system’s functionality using questionnaires. Results highlight the ease of use and motivational potential, with positive therapist feedback highlighting its customizability for therapy.

##### Cobot Control with Other Forms of Feedback

Various techniques, including dynamic movement primitives (DMPs), computational modeling, and self-learning methods, have also been used to control and adapt cobots for therapeutic purposes.

A study used a UR5 cobot with a force sensor, wrist support, custom handle, and force feedback to enhance training exercises [39]. The cobot used DMPs to learn exercise trajectories from demonstrations provided by therapists and personalized them based on force feedback. Simulated experiments showed the UR5’s potential for personalized rehabilitation.

A UR3 cobot, equipped with a 3D-printed cone-shaped tool and a force sensor, was programmed for real-time monitoring and intelligent self-learning control [41]. During the experiment, the cobot applied consistent resistive forces across various axes to enhance muscle engagement in a healthy participant. The results highlighted the system’s ability to adapt to varying user forces, suggesting its potential to improve rehabilitation outcomes.

A different study used the Franka Emika Panda cobot to generate reference paths by analyzing users’ motions [61]. With a custom-made handle, it learned and imitated user trajectories via an attention-based model, creating adaptive paths for ADL tasks. During tests with 10 healthy participants, the cobot adjusted in real-time to match user motions, suggesting the model could potentially enhance rehabilitation outcomes through individualized, assist-as-needed mode of assistance.

In another study, the authors proposed a teleoperation framework for remotely controlling a Sawyer robot in passive and active rehabilitation training for individuals with upper extremity hemiplegia [65]. Using a haptic device for therapist control, the system incorporated autonomous and interactive modes with improved DMPs, virtual fixtures, and a hybrid control strategy driven by sEMG-based forearm muscle activation. Two experiments validated the framework: in the first, a therapist remotely controlled the robot to collect demonstration trajectory data, while in the second, the robot autonomously followed a circular trajectory to test the motion model and control strategy. Results showed smooth trajectory reproduction without abrupt stops and effective generation of guiding virtual forces. These findings highlight the potential benefits of cobot-assisted rehabilitation.

An uncertainty compensated high-order adaptive iteration learning control (UCHAILC) method was developed to improve the tracking performance of a KUKA LBR iiwa R700 cobot during upper extremity rehabilitation for individuals with stroke [52]. The cobot was configured to provide assistance during ADLs. Hand movements during a drinking task were recorded from healthy participants using motion capture to generate end-effector trajectories. The results showed improved tracking accuracy, enabling more accurate and personalized assistance. This approach may improve rehabilitation outcomes by offering task-specific, repetitive training aligned with individual patient needs.

Another study used the Kuka LWR 4+ cobot and M-IMUs to develop an adaptive control system for patient rehabilitation [50]. This system, comprised of wrist support with a magnet and two M-IMU sensors, tracked arm movements and adjusted stiffness accordingly. Two healthy participants performed 2D and 3D point-to-point movements, replicating healthy behaviors and simulating post-stroke-like movements/behavior, such as failing to extend the elbow, moving in the wrong direction, or pausing during execution, with visual feedback in the form of pictures illustrating the tasks to be performed. The findings suggest that the system can safely adjust to individual needs and enhance patient engagement and therapy outcomes, thus indicating its potential for personalized rehabilitation.

In the article by Chiriatti et al. [46], the authors presented a framework using a UR5e cobot to assist users in executing specific 3D trajectories. A specialized handle, designed for individuals with limited grip, facilitated user interaction, while the developed algorithm applied elastic corrective forces to ensure linear movement and prevent deviations. The experimental procedure involves caregiver-defined start and end points, with the endpoint tracked by a camera and exercises initiated by sufficient user-applied force. Preliminary tests with healthy participants indicated that the system is intuitive and user-friendly. Safety measures, including speed limitation and seating outside the cobot’s reach, were implemented. Further trials with individuals with stroke will evaluate its efficacy.

#### Bilateral Upper Extremity Rehabilitation

Simultaneous use of cobots, specifically the UR5 and UR10 models, have been explored for bilateral upper extremity rehabilitation. A three-stage trajectory generation method was developed and assessed using these cobots [44]. The three stages are (1) workspace analysis of the intersection between the user and robot hands, (2) generation of personalized trajectories within the user-specific workspace, and (3) interference analysis to ensure training safety. Custom handles were used to facilitate training, and seven healthy participants completed eight predefined trajectory training sessions, demonstrating the method’s effectiveness for safe and individualized rehabilitation.

The system used in the article by Miao et al. [44] was further enhanced with force sensors, custom handlebars, and a Velcro$\circledR$-secured hand and wrist support [43]. The UR10 functioned as the master and the UR5 as the slave, employing a patient-cooperative control strategy for passive, active, and self-training assistance. Ten healthy participants performed shoulder flexion, extension, adduction, abduction, self-mimic, and self-cooperative exercises, showing that the cobots could provide a reliable bilateral training environment suitable for clinical use.

### Lower extremity rehabilitation

#### Cobot Control with Electrophysiological Data

A study developed a hybrid robotic rehabilitation system that integrated a ROBERT$\circledR$ with sEMG-triggered functional electrical stimulation (FES) for lower extremity rehabilitation [38]. The cobot (Kuka LBR), equipped with a leg brace and electrodes placed on the knee and foot extensor muscles, guided participants through predefined leg press and dorsiflexion trajectories. Ten healthy participants performed 40 repetitions at two sEMG thresholds, with FES triggered when sEMG signals exceeded preset thresholds and stopped at the trajectory endpoint. Results showed high success rates in exercise repetitions and force generation, suggesting that combining sEMG-triggered FES with robotic assistance may enhance rehabilitation outcomes.

Similarly, Leerskov et al. [59] developed a hybrid system combining ROBERT$\circledR$ with FES for lower extremity rehabilitation. The system included a custom-made brace, stimulation electrodes placed over the knee flexor and extensor muscles, and EMG electrodes to monitor muscle activity. The robot adjusted resistive forces and stimulation intensity based on individual needs, with FES administered by an experimenter, which automatically stopped at 80% of the trajectory. Eight participants completed 50 FES-assisted leg-press repetitions and showed variability in outcomes—half exhibited potentiation (increased velocity and force), and half experienced fatigue. These findings underscore the need for adaptive rehabilitation systems that can address the diverse patient responses.

The feasibility of a UR10e cobot for lower extremity rehabilitation was evaluated, with its performance compared to that of a physiotherapist [48]. The cobot was equipped with a custom-designed brace attached to its final joint, securely fastened to the user’s lower extremity for therapeutic movements. It was programmed to perform repetitive movements based on Proprioceptive Neuromuscular Facilitation techniques, customized to the user’s leg positioning. Inertia measurement unit sensors tracked pelvic and lower leg movements during passive and assistive training modes. The results showed the cobot’s effectiveness in performing repeatable exercises, though accuracy depended on the trajectory programming. These findings highlight the potential cost and efficiency benefits of cobot-assisted rehabilitation.

#### Cobot Control with Assist-As-Needed (AAN) Strategy

Leerskov et al. improved the system in [59] by integrating ROBERT$\circledR$ with FES and an AAN strategy for lower extremity stroke rehabilitation [58]. A foot brace with EMG and stimulation electrodes placed on the thigh and the tibialis anterior allowed adaptive support for ankle dorsiflexion and knee extension based on user capabilities. Assistance modes, including no support, FES, mechanical assistance, or both, were dynamically adjusted using EMG signals to detect voluntary effort and trigger the AAN system. Tests with 10 healthy participants showed over 96% accuracy in detecting user behavior and adjusting assistance levels. Clinical feasibility with two individuals post-stroke indicated enhanced engagement, voluntary effort, and potential for motor learning. These findings suggest promise for personalized stroke rehabilitation, although further clinical trials are needed.

Building upon their previous work described in [58], the authors in [57] assessed the feasibility of combining ROBERT$\circledR$ and FES with an AAN approach to support actively initiated leg movements in individuals with stroke. Using the previously described cobot in [58], assistance levels were categorized from the patient’s perspective as no assistance or assistance, with the latter further subdivided into FES alone or combined FES with mechanical support. Nine individuals with subacute stroke performed repetitive ankle dorsiflexion and knee extension movements, with and without assistance. The results showed that assistance was required in 44% of ankle dorsiflexion repetitions and 5% of knee extension repetitions, and median fatigue scores indicated mild-to-moderate perceived fatigue. The findings suggest that integrating robotics and FES within an AAN approach is feasible for supporting leg movements in stroke rehabilitation.

#### Cobot Control with Other Forms of Feedback

The Kuka LBR iiwa was investigated for ankle rehabilitation using a custom 3D-printed leg-constraining and sole plates for secure engagement during therapy [32]. Five healthy participants performed 20-min predefined movements. The results highlight the cobot for effective ankle rehabilitation, but further development is needed to enhance movement control and monitoring.

Similarly, another study evaluated the feasibility of a large-scale trial to assess ROBERT$\circledR$’s effectiveness in improving hip flexor strength after SCI [56]. A leg brace attached to robot’s end effector provided guidance or active assistance depending on participant’s muscle strength grades. Four participants with subacute SCI and hip flexor muscle strength grades between 1 and 3 performed 60 hip flexion repetitions on one leg three times weekly for 4 weeks, while the other leg served as a control. Results demonstrated 92% training adherence, no adverse events, and positive feedback, suggesting that ROBERT$\circledR$ is acceptable and potentially effective. These findings indicate that cobot-assisted training is feasible for enhancing hip flexor strength, although further research is needed to improve the system.

Another study analyzed the cost-effectiveness of the ROBERT$$\circledR$$ device for lower extremity therapy compared to physiotherapist-led sessions [60]. With an average cost below USD $25 per hour, ROBERT$$\circledR$$ demonstrated the potential to reduce hospital stays, readmission rates, and overall healthcare expenses. Despite its high initial capital cost, the system’s operating expenses were only one-tenth that of a specialty outpatient session in Hong Kong hospitals. This suggests that ROBERT$$\circledR$$ offers a cost-effective solution for optimizing rehabilitation costs in clinical settings.

## Discussion

This systematic narrative review explored the use of industrial-grade cobots for upper and lower extremity motor rehabilitation among individuals with stroke and SCI. Cobots offer unique advantages such as advanced safety features, compliant actuators, and real-time adaptability, which allow safe and interactive human–robot collaboration without physical barriers. These characteristics make cobots a promising tool for rehabilitation, addressing some limitations of traditional robots that often lack flexibility and require external force-sensing modules.

Few studies included individuals with stroke or SCI, primarily focusing on lower extremity rehabilitation using the ROBERT$\circledR$ system. Robotic assistance with or without AAN strategies and/or FES demonstrated improvements in patient engagement, voluntary effort, and task-specific motor recovery [56–58]. One study reported that participants achieved 381 repetitions with AAN activated compared to 35 without it, highlighting the system’s capacity to enhance engagement and rehabilitation dose through increased repetition [57].

Voluntary effort was further emphasized in trials involving the ROBERT$\circledR$ device, as participants actively contributed to their movements, supporting guided assistance in motor learning [58]. Participants expressed higher motivation and perceived robotic assistance as more challenging than conventional physiotherapy, suggesting that cobot-assisted rehabilitation may foster involvement and effort [56].

Repetitive movement practice may facilitate task-specific motor recovery by targeting muscle activation and functional improvement. The AAN algorithm dynamically adjusted support, encouraging patients to perform more tasks independently, leveraging residual motor capacity and promoting neuroplasticity [58].

Despite these promising outcomes, the absence of research on cobots for upper extremity rehabilitation in stroke and SCI populations highlights a critical gap. Expanding studies to upper extremity rehabilitation will be fundamental to exploring the full therapeutic potential of cobots for diverse motor impairments.

The collaborative design of cobots makes them well-suited for clinical settings, as they enable the delivery of repetitive, task-specific exercises, which are essential for motor recovery among individuals with stroke and SCI. Unlike exoskeletons that require precise alignment and often involve complex setup procedures, or end-effector robots that are limited to planar movements, cobots can adapt to various therapeutic interventions and can support complex, three-dimensional exercises with customizable tools. Their adaptability in reach (ranging from 500 mm to 1300 mm) and payload capabilities (ranging from 0.5 kg to 10 kg) allow them to meet the diverse needs of patients for upper extremity rehabilitation. However, for lower extremity rehabilitation, cobots are limited to mobilization exercises usually performed in the supine position, which, unlike exoskeletons or foot-plate-based end effector robots, do not provide functional training necessary for standing or walking. This distinction highlights the supplementary role cobots play in lower extremity rehabilitation.

Cobots show potential for enhancing patient engagement in rehabilitation through strategies such as gamification, virtual reality, and task-specific exercises [58]. Studies in this review integrated technologies like virtual reality, sEMG monitoring, and adjustable trajectories to create interactive and motivating therapy experiences. These approaches not only align with conventional rehabilitation objectives but also introduce novel ways to improve adherence. By tailoring assistance levels to individual requirements, cobots offer personalized therapy comparable to conventional methods. Several studies implemented AAN control strategies to dynamically adjust robotic assistance based on user effort. Assist-as-needed systems can promote active participation, improve movement smoothness, and enhance motor recovery by providing personalized support. Although promising, further clinical studies are required to validate their efficacy across diverse patient populations with motor impairments.

Cost-effectiveness is a key consideration for implementing cobots in rehabilitation settings. Although this narrative review did not address the cost-effectiveness of cobots in rehabilitation settings, preliminary studies suggest that systems, such as ROBERT$\circledR$, used for lower extremity therapy, may reduce healthcare costs by decreasing hospital stays and readmission rates [48, 60]. However, high initial investment and variability in operational expenses highlight the need for more comprehensive research to determine long-term cost-effectiveness compared to conventional therapy. Future studies should address this gap to inform the implementation and scalability of cobots in clinical practice.

Despite these promising findings, several challenges remain in current research. Successful integration into clinical practice requires continuous training and support for therapists to operate and maximize the benefits of these technologies [70, 71].

Furthermore, there are inconsistencies in the definitions of cobots and traditional industrial robots, creating ambiguity in the field. Many studies focus on methodologies, lack sufficient details about the cobot characteristics, and have limited information regarding the technical specifications of the cobots used. Additionally, the low payload of some cobots may restrict their ability to provide personalized exercises requiring gravity compensation. Addressing these gaps is crucial to advancing cobot’s applicability in rehabilitation.

## Conclusion

This review highlights the emerging potential of cobots in motor rehabilitation after a stroke or SCI. While cobots present significant potential for motor rehabilitation of the upper and lower extremities among individuals with stroke or SCI, further research is needed to optimize their integration in rehabilitation settings. Standardizing definitions, improving research methodologies, and conducting studies involving more stroke and SCI populations will enhance the generalizability of the results. Addressing these challenges may further refine the effectiveness of cobots in rehabilitation and support their sustainable integration into clinical practice. 

## Supplementary Information

Supplementary Material 1.

## Data Availability

Not applicable.

## Abbreviations

AAN: Assist-as-needed
ADLs: Activities of daily living
Cobots: Collaborative robots
DMPs: Dynamic movement primitives
DOFs: Degrees of freedom
IMU: Inertial measurement unit
M-IMU: Magneto-inertial measurement units
MIME: Mirror Image movement enabler
SCI: Spinal cord injury
sEMG: Surface electromyography
UR: Universal Robots
VR: Virtual reality
