# Supplementary material for: Industrial-grade collaborative robots for motor rehabilitation after stroke and spinal cord injury: a systematic narrative review
Source: Biomed Eng Online. 2025 Apr 30;24:50. doi: 10.1186/s12938-025-01362-z (PMC12042587; doi:10.1186/s12938-025-01362-z)
Supplement: Supplementary file 1 — Supplementary Material 1. [file 12938_2025_1362_MOESM1_ESM.pdf]

Appendix A Search Strategy

Database: Ovid MEDLINE(R) ALL (1946 to November 28, 2024)
Platform: Ovid
Date last searched November 28, 2024

1 (collab* adj3 robot*).tw,kf.
2 (cooperat* adj3 robot*).tw,kf.
3 (co-bot* or cobot*).tw,kf.
4 movement? primitive?.tw,kf.
5 (automat* adj1 Robot*).tw,kf.
6 ((human? or patient? or person?) adj2 robot* adj2 (co-manipulat* or comanipulat* or collaboration? or cooperation or co-operation or interaction? or "shared space" or "sharing space")).tw,kf.
7 (industrial* adj1 Robot*).tw,kf.
8 (reprogram* adj1 Robot*).tw,kf.
9 (re-program* adj1 Robot*).tw,kf.
10 (reconfig* adj1 Robot*).tw,kf.
11 (re-config* adj1 Robot*).tw,kf.
12 (UR3 or UR10 or UR5 or "Universal Robot* 5" or "Universal Robot* 10" or "Universal Robot* 3" or "Franka Emika" or "KUKA LBR IIWA" or "Yumi robot").tw,kf.
13 Intelligent Assist Device?.tw,kf.
14 assist as needed.tw,kf.
15 (hybrid* adj3 robot*).tw,kf.
16 (ROBERT and robot*).tw,kf.
17 or/1-16
18 Robotics/
19 Man-Machine Systems/
20 18 and 19
21 17 or 20
22 "Physical and Rehabilitation Medicine"/
23 exp rehabilitation/
24 rehab*.tw,kf,jw.
25 telerehab*.tw,kf,jw.
26 neurorehab*.tw,kf,jw.
27 rh.fs.

28 Rehabilitation Centers/
29 occupational therapy/
30 (occupational adj therap*).tw,kf,jw.
31 physical therapy specialty/
32 (physical adj therap*).tw,kf,jw.
33 physiotherap*.tw,kf,jw.
34 physio-therapist*.tw,kf,jw.
35 motor learn*.tw,kf.
36 motor relearn*.tw,kf.
37 motor re-learn*.tw,kf.
38 (treatment* or training).tw,kf.
39 Exercise Movement Techniques/
40 exp Exercise/
41 exp Exercise Therapy/
42 exercis*.tw,kf.
43 (intervention adj2 (robot* or mobilit*)).tw,kf.
44 or/22-43
45 21 and 44
46 Cerebrovascular disorders/
47 exp Stroke/
48 Stroke Rehabilitation/
49 (stroke or strokes or poststroke or poststrokes or apoplex* or cerebral vasc* or cerebrovasc* or cva or tia or transient isch?emic attack*).tw,kf.
50 ((brain? or cerebr* or cerebell* or spinal cord or vertebrobasil* or hemispher* or intracran* or intracerebral or infratentorial or supratentorial or mca* or anterior circulat* or middle circulat* or posterior circulat*) adj3 (isch?emi* or infarct* or thrombo* or emboli* or occlus* or hypoxi*)).tw,kf.
51 ((brain? or cerebr* or cerebell* or spinal cord or vertebrobasil* or hemispher* or intracran* or intracerebral or infratentorial or supratentorial or mca* or anterior circulat* or middle circulat* or posterior circulat*) adj3 (haemorrhag* or hemorrhag* or haematoma* or hematoma* or bleed*)).tw,kf.
52 Hemiplegia/
53 exp Gait Disorders, Neurologic/
54 (hemipleg* or hemipar* or paresis or paretic).tw,kf.
55 exp Paresis/
56 exp Spinal Cord Injuries/
57 exp Spinal Cord Ischemia/
58 exp Central Cord Syndrome/
59 (myelopathy adj3 (traumatic or post-traumatic)).tw,kf.
60 ((spine or spinal) adj3 (fracture* or wound* or trauma* or injur* or damag* or surg* or fusion*)).tw,kf.
61 (spinal cord adj3 (contusion* or laceration* or transaction* or trauma* or ischemi*)).tw,kf.
62 SCI.tw,kf.
63 exp Paraplegia/

64 exp Quadriplegia/
65 (paraplegia* or quadriplegia* or tetraplegia*).tw,kf.
66 Spinal Cord Compression/
67 exp Spine/in
68 exp Spine/su
69 central spinal cord syndrome.tw,kf.
70 central cord injury syndrome.tw,kf.
71 exp Upper Extremity/
72 (upper limb* or upper extremit* or arm* or shoulder* or hand* or axilla* or elbow* or forearm* or finger* or wrist*).tw,kf.
73 (dexterity or dexterous*).tw,kf.
74 (grip or gripping).tw,kf.
75 (grasp or grasping).tw,kf.
76 (tap or tapping).tw,kf.
77 (reach or reaching).tw,kf.
78 exp Lower Extremity/
79 (lower limb* or lower extremit* or leg or legs or foot or feet or thigh* or knee* or ankle* or balance or gait* or walk* or ambulat* or stabilit* or mobilit* or locomot* or step* or stride or strides).tw,kf.
80 or/46-79
81 45 and 80
82 limit 81 to english language

Database: Cochrane Central Register of Controlled Trials (2014 to Present)
Date last searched: November 28, 2024
Platform: Ovid
1 (collab* adj3 robot*).tw,kf.
2 (cooperat* adj3 robot*).tw,kf.
3 (co-bot* or cobot*).tw,kf.
4 movement? primitive?.tw,kf.
5 (automat* adj1 Robot*).tw,kf.
6 ((human? or patient? or person?) adj2 robot* adj2 (co-manipulat* or comanipulat* or collaboration? or cooperation or co-operation or interaction? or "shared space" or "sharing space")).tw,kf.
7 (industrial* adj1 Robot*).tw,kf.
8 (reprogram* adj1 Robot*).tw,kf.
9 (re-program* adj1 Robot*).tw,kf.
10 (reconfig* adj1 Robot*).tw,kf.
11 (re-config* adj1 Robot*).tw,kf.
12 (UR3 or UR10 or UR5 or "Universal Robot* 5" or "Universal Robot* 10" or "Universal Robot* 3" or "Franka Emika" or "KUKA LBR IIWA" or "Yumi robot").tw,kf.
13 Intelligent Assist Device?.tw,kf.
14 assist as needed.tw,kw.
15 (hybrid* adj3 robot*).tw,kw.
16 (ROBERT and robot*).tw,kw.

17 or/1-16
18 Robotics/
19 Man-Machine Systems/
20 18 and 19
21 17 or 20
22 "Physical and Rehabilitation Medicine"/
23 exp rehabilitation/
24 rehab*.tw,kf,jw.
25 telerehab*.tw,kf,jw.
26 neurorehab*.tw,kf,jw.
27 rh.fs.
28 Rehabilitation Centers/
29 occupational therapy/
30 (occupational adj therap*).tw,kf,jw.
31 physical therapy specialty/
32 (physical adj therap*).tw,kf,jw.
33 physiotherap*.tw,kf,jw.
34 physio-therapist*.tw,kf,jw.
35 motor learn*.tw,kf.
36 motor relearn*.tw,kf.
37 motor re-learn*.tw,kf.
38 (treatment* or training).tw,kf.
39 Exercise Movement Techniques/
40 exp Exercise/
41 exp Exercise Therapy/
42 exercis*.tw,kf.
43 (intervention adj2 (robot* or mobilit*)).tw,kf.
44 or/22-43
45 21 and 44
46 Cerebrovascular disorders/
47 exp Stroke/
48 Stroke Rehabilitation/
49 (stroke or strokes or poststroke or poststrokes or apoplex* or cerebral vasc* or cerebrovasc* or cva or tia or transient isch?emic attack*).tw,kf.
50 ((brain? or cerebr* or cerebell* or spinal cord or vertebrobasil* or hemispher* or intracran* or intracerebral or infratentorial or supratentorial or mca* or anterior circulat* or middle circulat* or posterior circulat*) adj3 (isch?emi* or infarct* or thrombo* or emboli* or occlus* or hypoxi*)).tw,kf.
51 ((brain? or cerebr* or cerebell* or spinal cord or vertebrobasil* or hemispher* or intracran* or intracerebral or infratentorial or supratentorial or mca* or anterior circulat* or middle circulat* or posterior circulat*) adj3 (haemorrhag* or hemorrhag* or haematoma* or hematoma* or bleed*)).tw,kf.
52 Hemiplegia/
53 exp Gait Disorders, Neurologic/
54 (hemipleg* or hemipar* or paresis or paretic).tw,kf.

55 exp Paresis/
56 exp Spinal Cord Injuries/
57 exp Spinal Cord Ischemia/
58 exp Central Cord Syndrome/
59 (myelopathy adj3 (traumatic or post-traumatic)).tw,kf.
60 ((spine or spinal) adj3 (fracture* or wound* or trauma* or injur* or damag* or surg* or fusion*)).tw,kf.
61 (spinal cord adj3 (contusion* or laceration* or transaction* or trauma* or ischemi*)).tw,kf.
62 SCI.tw,kf.
63 exp Paraplegia/
64 exp Quadriplegia/
65 (paraplegia* or quadriplegia* or tetraplegia*).tw,kf.
66 Spinal Cord Compression/
67 exp Spine/in
68 exp Spine/su
69 central spinal cord syndrome.tw,kf.
70 central cord injury syndrome.tw,kf.
71 exp Upper Extremity/
72 (upper limb* or upper extremit* or arm* or shoulder* or hand* or axilla* or elbow* or forearm* or finger* or wrist*).tw,kf.
73 (dexterity or dexterous*).tw,kf.
74 (grip or gripping).tw,kf.
75 (grasp or grasping).tw,kf.
76 (tap or tapping).tw,kf.
77 (reach or reaching).tw,kf.
78 exp Lower Extremity/
79 (lower limb* or lower extremit* or leg or legs or foot or feet or thigh* or knee* or ankle* or balance or gait* or walk* or ambulat* or stabilit* or mobilit* or locomot* or step* or stride or strides).tw,kf.
80 or/46-79
81 45 and 80
82 limit 81 to english language
83 trial registry record.pt.
84 82 not 83

Database: Embase (1974 to 2024 November 28)
Platform: Ovid
Date last searched November 28, 2024
1 (collab* adj3 robot*).tw,kf.
2 (cooperat* adj3 robot*).tw,kf.
3 (co-bot* or cobot*).tw,kf.
4 movement? primitive?.tw,kf.
5 (automat* adj1 Robot*).tw,kf.
6 ((human? or patient? or person?) adj2 robot* adj2 (co-manipulat* or comanipulat*

or collaboration? or cooperation or co-operation or interaction? or ”shared space” or ”sharing space”)).tw,kf.
7 (industrial* adj1 Robot*).tw,kf.
8 (reprogram* adj1 Robot*).tw,kf.
9 (re-program* adj1 Robot*).tw,kf.
10 (reconfig* adj1 Robot*).tw,kf.
11 (re-config* adj1 Robot*).tw,kf.
12 (UR3 or UR10 or UR5 or ”Universal Robot* 5” or ”Universal Robot* 10” or ”Universal Robot* 3” or ”Franka Emika” or ”KUKA LBR IIWA” or ”Yumi robot”).tw,kf.
13 Intelligent Assist Device?.tw,kf.
14 assist as needed.tw,kf.
15 (hybrid* adj3 robot*).tw,kf.
16 (ROBERT and robot*).tw,kf.
17 or/1-16
18 robotics/
19 man machine interaction/
20 18 and 19
21 17 or 20
22 rehabilitation medicine/ or physical medicine/
23 exp rehabilitation/
24 rehab*.tw,kf,jx.
25 telerehab*.tw,kf,jx.
26 neurorehab*.tw,kf,jx.
27 rh.fs.
28 Rehabilitation Center/
29 occupational therapy/
30 (occupational adj therap*).tw,kf,jx.
31 exp physiotherapy/
32 (physical adj therap*).tw,kf,jx.
33 physiotherap*.tw,kf,jx.
34 physio-therapist*.tw,kf,jx.
35 motor learn*.tw,kf.
36 motor relearn*.tw,kf.
37 motor re-learn*.tw,kf.
38 (treatment* or training).tw,kf.
39 Exercise Movement Techniques/
40 exp exercise/
41 exp kinesiotherapy/
42 exercis*.tw,kf.
43 (intervention adj2 (robot* or mobilit*)).tw,kf.
44 or/22-43
45 21 and 44
46 cerebrovascular disease/
47 exp cerebrovascular accident/
48 stroke rehabilitation/

49 (stroke or strokes or poststroke or poststrokes or apoplex* or cerebral vasc* or cerebrovasc* or cva or tia or transient isch?emic attack*).tw,kf.
50 ((brain? or cerebr* or cerebell* or spinal cord or vertebrobasil* or hemispher* or intracran* or intracerebral or infratentorial or supratentorial or mca* or anterior circulat* or middle circulat* or posterior circulat*) adj3 (isch?emi* or infarct* or thrombo* or emboli* or occlus* or hypoxi*)).tw,kf.
51 ((brain? or cerebr* or cerebell* or spinal cord or vertebrobasil* or hemispher* or intracran* or intracerebral or infratentorial or supratentorial or mca* or anterior circulat* or middle circulat* or posterior circulat*) adj3 (haemorrhag* or hemorrhag* or haematoma* or hematoma* or bleed*)).tw,kf.
52 hemiplegia/
53 exp neurologic gait disorder/
54 (hemipleg* or hemipar* or paresis or paretic).tw,kf.
55 exp paralysis/
56 exp spinal cord injury/
57 spinal cord ischemia/
58 (myelopathy adj3 (traumatic or post-traumatic)).tw,kf. 183
59 ((spine or spinal) adj3 (fracture* or wound* or trauma* or injur* or damag* or surg* or fusion*)).tw,kf.
60 (spinal cord adj3 (contusion* or laceration* or transaction* or trauma* or ischemi*)).tw,kf. 61 SCI.tw,kf.
62 paraplegia/
63 quadriplegia/
64 (paraplegia* or quadriplegia* or tetraplegia*).tw,kf.
65 central spinal cord syndrome.tw,kf.
66 central cord injury syndrome.tw,kf.
67 exp upper limb/
68 (upper limb* or upper extremit* or arm* or shoulder* or hand* or axilla* or elbow* or forearm* or finger* or wrist*).tw,kf.
69 (dexterity or dexterous*).tw,kf.
70 (grip or gripping).tw,kf.
71 (grasp or grasping).tw,kf.
72 (tap or tapping).tw,kf.
73 (reach or reaching).tw,kf.
74 exp lower limb/
75 (lower limb* or lower extremit* or leg or legs or foot or feet or thigh* or knee* or ankle* or balance or gait* or walk* or ambulat* or stabilit* or mobilit* or locomot* or step* or stride or strides).tw,kf.
76 or/46-75
77 45 and 76
78 limit 77 to english language

Database: Web of Science Core Collection
Platform: N/A
Date last searched November 28, 2024
1: (TS=(co-bot? or cobot?) OR TS=(cooperat* NEAR/3 robot*) OR TS=(collab* NEAR/3 robot*) OR TS=("movement* primitive*") OR TS=(automat* NEAR/1 Robot*) OR TS=(industrial* NEAR/1 Robot*) OR TS=(reprogram* NEAR/1 Robot*) OR TS=(re-program* NEAR/1 Robot*) OR TS=(reconfig* NEAR/1 Robot*) OR TS=(re-config* NEAR/1 Robot*) OR TS=("human-robot" NEAR/2 (collaboration? or cooperation or interaction? OR "shared space" OR "sharing space")) OR TS=("patient-robot" NEAR/2 (collaboration? or cooperation or interaction? OR "shared space" OR "sharing space")) OR TS=("person-robot" NEAR/2 (collaboration? or cooperation or interaction? OR "shared space" OR "sharing space")) OR TS=(UR3 OR UR10 OR UR5 OR "Universal Robot* 5" OR "Universal Robot* 10" OR "Universal Robot* 3" OR "Franka Emika" OR "KUKA LBR IIWA" OR "Yumi robot") OR TS=("Intelligent Assist Device" OR "intelligent assist devices") OR TS=("assist as needed" OR (hybrid* NEAR/3 robot*) OR (ROBERT AND robot*)))

2: (ALL=(rehab* OR telerehab* OR neurorehab* OR "physical therap*" OR physiotherap* OR "physio-therap*" OR "occupational therap*") OR TS=("motor relearn*" OR "motor re-learn*" or "motor learn*" or treatment or training or exercis*) OR TS=(intervention NEAR/2 (robot* or mobilit*)))

3: TS=(stroke or strokes or poststroke or poststrokes or hemipleg* or hemipar* OR paresis or paretic or apoplex* or "cerebral vasc*" or cerebrovasc* or cva or tia or "transient ischemic attack" or "transient ischaemic attack" OR SCI OR spine OR spinal OR parapleg* or tetrapleg* or quadripleg* OR "upper limb*" OR "upper extremit*" OR "lower limb*" or "lower extremit*" OR arm or arms or shoulder or shoulders or hand or hands or axilla or elbow or elbows or forearm or forearms or finger or fingers or wrist or wrists OR dexterity OR dextrous OR grip OR gripping OR grasp OR grasping OR tap OR tapping OR reach OR reaching OR leg OR legs OR foot OR feet OR thigh* OR knee* OR ankle* OR balance OR gait* OR walk* OR ambulat* OR stabilit* OR mobilit* OR locomot* OR step* OR stride OR strides) OR TS=((brain) NEAR/3 (ischemi* or ischaemi* or infarct* or thrombo* or emboli* or occlus* or hypoxi* or haemorrhag* or hemorrhag* or haematoma* or hematoma* or bleed*)) OR TS=((cerebr*) NEAR/3 (ischemi* or ischaemi* or infarct* or thrombo* or emboli* or occlus* or hypoxi* or haemorrhag* or hemorrhag* or haematoma* or hematoma* or bleed*)) OR TS=((cerebell*) NEAR/3 (ischemi* or ischaemi* or infarct* or thrombo* or emboli* or occlus* or hypoxi* or haemorrhag* or hemorrhag* or haematoma* or hematoma* or bleed*)) OR TS=((vertebrobasil*) NEAR/3 (ischemi* or ischaemi* or infarct* or thrombo* or emboli* or occlus* or hypoxi* or haemorrhag* or hemorrhag* or haematoma* or hematoma* or bleed*)) OR TS=((hemispher*) NEAR/3 (ischemi* or ischaemi* or infarct* or thrombo* or emboli* or occlus* or hypoxi* or haemorrhag* or hemorrhag* or haematoma* or

hematoma* or bleed*)) OR TS=((intracran*) NEAR/3 (ischemi* or ischaemi* or infarct* or thrombo* or emboli* or occlus* or hypoxi* or haemorrhag* or hemorrhag* or haematoma* or hematoma* or bleed*)) OR TS=((intracerebral) NEAR/3 (ischemi* or ischaemi* or infarct* or thrombo* or emboli* or occlus* or hypoxi* or haemorrhag* or hemorrhag* or haematoma* or hematoma* or bleed*)) OR TS=((infratentorial) NEAR/3 (ischemi* or ischaemi* or infarct* or thrombo* or emboli* or occlus* or hypoxi* or haemorrhag* or hemorrhag* or haematoma* or hematoma* or bleed*)) OR TS=((supratentorial) NEAR/3 (ischemi* or ischaemi* or infarct* or thrombo* or emboli* or occlus* or hypoxi* or haemorrhag* or hemorrhag* or haematoma* or hematoma* or bleed*)) OR TS=((mca*) NEAR/3 (ischemi* or ischaemi* or infarct* or thrombo* or emboli* or occlus* or hypoxi* or haemorrhag* or hemorrhag* or haematoma* or hematoma* or bleed*)) OR TS=((”anterior circulat*”) NEAR/3 (ischemi* or ischaemi* or infarct* or thrombo* or emboli* or occlus* or hypoxi* or haemorrhag* or hemorrhag* or haematoma* or hematoma* or bleed*)) OR TS=((”middle circulat*”) NEAR/3 (ischemi* or ischaemi* or infarct* or thrombo* or emboli* or occlus* or hypoxi* or haemorrhag* or hemorrhag* or haematoma* or hematoma* or bleed*)) OR TS=((”posterior circulat*”) NEAR/3 (ischemi* or ischaemi* or infarct* or thrombo* or emboli* or occlus* or hypoxi* or haemorrhag* or hemorrhag* or haematoma* or hematoma* or bleed*))
4: #1 AND #2 AND #3

5: #1 AND #2 AND #3 and English (Languages)

Database: Scopus
Platform: N/A
Date last searched November 28, 2024

(TITLE-ABS-KEY(stroke or strokes or poststroke or poststrokes or hemipleg* or hemipar* OR paresis or paretic or apoplex* or ”cerebral vasc*” or cerebrovasc* or cva or tia or ”transient ischemic attack” or ”transient ischaemic attack” OR SCI OR spine OR spinal OR parapleg* or tetrapleg* or quadripleg* OR ”upper limb*” OR ”upper extremit*” OR ”lower limb*” or ”lower extremit*” OR arm or arms or shoulder or shoulders or hand or hands or axilla or elbow or elbows or forearm or forearms or finger or fingers or wrist or wrists OR dexterity OR dextrous OR grip OR gripping OR grasp OR grasping OR tap OR tapping OR reach OR reaching OR leg OR legs OR foot OR feet OR thigh* OR knee* OR ankle* OR balance OR gait* OR walk* OR ambulat* OR stabilit* OR mobilit* OR locomot* OR step* OR stride OR strides) OR TITLE-ABS-KEY((brain) W/3 (ischemi* or ischaemi* or infarct* or thrombo* or emboli* or occlus* or hypoxi* or haemorrhag* or hemorrhag* or haematoma* or hematoma* or bleed*)) OR TITLE-ABS-KEY((cerebr*) W/3 (ischemi* or ischaemi* or infarct* or thrombo* or emboli* or occlus* or hypoxi* or haemorrhag* or hemorrhag* or haematoma* or hematoma* or bleed*)) OR TITLE-ABS-KEY((cerebell*) W/3 (ischemi* or ischaemi* or infarct* or thrombo* or emboli* or occlus* or hypoxi* or haemorrhag* or hemorrhag* or haematoma* or hematoma*

or bleed*)) OR TITLE-ABS-KEY((vertebrobasil*) W/3 (ischemi* or ischaemi* or infarct* or thrombo* or emboli* or occlus* or hypoxi* or haemorrhag* or hemorrhag* or haematoma* or hematoma* or bleed*)) OR TITLE-ABS-KEY((hemispher*) W/3 (ischemi* or ischaemi* or infarct* or thrombo* or emboli* or occlus* or hypoxi* or haemorrhag* or hemorrhag* or haematoma* or hematoma* or bleed*)) OR TITLE-ABS-KEY((intracran*) W/3 (ischemi* or ischaemi* or infarct* or thrombo* or emboli* or occlus* or hypoxi* or haemorrhag* or hemorrhag* or haematoma* or hematoma* or bleed*)) OR TITLE-ABS-KEY((intracerebral) W/3 (ischemi* or ischaemi* or infarct* or thrombo* or emboli* or occlus* or hypoxi* or haemorrhag* or hemorrhag* or haematoma* or hematoma* or bleed*)) OR TITLE-ABS-KEY((infratentorial) W/3 (ischemi* or ischaemi* or infarct* or thrombo* or emboli* or occlus* or hypoxi* or haemorrhag* or hemorrhag* or haematoma* or hematoma* or bleed*)) OR TITLE-ABS-KEY((supratentorial) W/3 (ischemi* or ischaemi* or infarct* or thrombo* or emboli* or occlus* or hypoxi* or haemorrhag* or hemorrhag* or haematoma* or hematoma* or bleed*)) OR TITLE-ABS-KEY((mca*) W/3 (ischemi* or ischaemi* or infarct* or thrombo* or emboli* or occlus* or hypoxi* or haemorrhag* or hemorrhag* or haematoma* or hematoma* or bleed*)) OR TITLE-ABS-KEY((”anterior circulat*”) W/3 (ischemi* or ischaemi* or infarct* or thrombo* or emboli* or occlus* or hypoxi* or haemorrhag* or hemorrhag* or haematoma* or hematoma* or bleed*)) OR TITLE-ABS-KEY((”middle circulat*”) W/3 (ischemi* or ischaemi* or infarct* or thrombo* or emboli* or occlus* or hypoxi* or haemorrhag* or hemorrhag* or haematoma* or hematoma* or bleed*)) OR TITLE-ABS-KEY((”posterior circulat*”) W/3 (ischemi* or ischaemi* or infarct* or thrombo* or emboli* or occlus* or hypoxi* or haemorrhag* or hemorrhag* or haematoma* or hematoma* or bleed*))) AND (ALL(rehab* OR telerehab* OR neurorehab* OR ”physical therap*” OR physiotherap* OR ”physio-therap*” OR ”occupational therap*”) OR TITLE-ABS-KEY(”motor relearn*” OR ”motor re-learn*” or ”motor learn*” or treatment or training or exercis*) OR TITLE-ABS-KEY(intervention W/2 (robot* or mobilit*))) AND ((TITLE-ABS-KEY(co-bot? or cobot?) OR TITLE-ABS-KEY(cooperat* W/3 robot*) OR TITLE-ABS-KEY(collab* W/3 robot*) OR TITLE-ABS-KEY(”movement* primitive*”) OR TITLE-ABS-KEY(automat* W/1 Robot*) OR TITLE-ABS-KEY(industrial* W/1 Robot*) OR TITLE-ABS-KEY(reprogram* W/1 Robot*) OR TITLE-ABS-KEY(re-program* W/1 Robot*) OR TITLE-ABS-KEY(reconfig* W/1 Robot*) OR TITLE-ABS-KEY(re-config* W/1 Robot*) OR TITLE-ABS-KEY(”human-robot” W/2 (collaboration? or cooperation or interaction? OR ”shared space” OR ”sharing space”)) OR TITLE-ABS-KEY(”patient-robot” W/2 (collaboration? or cooperation or interaction? OR ”shared space” OR ”sharing space”)) OR TITLE-ABS-KEY(”person-robot” W/2 (collaboration? or cooperation or interaction? OR ”shared space” OR ”sharing space”)) OR TITLE-ABS-KEY(UR3 OR UR10 OR UR5 OR ”Universal Robot* 5” OR ”Universal Robot* 10” OR ”Universal Robot* 3” OR ”Franka Emika” OR ”KUKA LBR IIWA” OR ”Yumi robot”) OR TITLE-ABS-KEY(”Intelligent Assist Device” OR ”intelligent assist devices”) OR TITLE-ABS-KEY(“assist as needed” OR (hybrid* W/3 robot*) OR (ROBERT AND robot*)))) AND (LIMIT-TO (LANGUAGE,”English”))

Database: Proquest Dissertations & Theses Global
Platform: ProQuest
Date last searched November 28, 2024

Set #1:
(NOFT((”middle circulat*”) NEAR/3 (ischemi* or ischaemi* or infarct* or thrombo* or emboli* or occlus* or hypoxi* or haemorrhag* or hemorrhag* or haematoma* or hematoma* or bleed*)) OR NOFT((”posterior circulat*”) NEAR/3 (ischemi* or ischaemi* or infarct* or thrombo* or emboli* or occlus* or hypoxi* or haemorrhag* or hemorrhag* or haematoma* or hematoma* or bleed*))) AND (NOFT(rehab* OR telerehab* OR neurorehab* OR ”physical therap*” OR physiotherap* OR ”physio-therap*” OR ”occupational therap*”) OR NOFT(”motor relearn*” OR ”motor re-learn*” or ”motor learn*” or treatment or training or exercis*) OR NOFT(intervention NEAR/2 (robot* or mobilit*))) AND ((NOFT(co-bot? or cobot?) OR NOFT(cooperat* NEAR/3 robot*) OR NOFT(collab* NEAR/3 robot*) OR NOFT(”movement* primitive*”) OR NOFT(automat* NEAR/1 Robot*) OR NOFT(industrial* NEAR/1 Robot*) OR NOFT(reprogram* NEAR/1 Robot*) OR NOFT(re-program* NEAR/1 Robot*) OR NOFT(reconfig* NEAR/1 Robot*) OR NOFT(re-config* NEAR/1 Robot*) OR NOFT(”human-robot” NEAR/2 (collaboration? or cooperation or interaction? OR ”shared space” OR ”sharing space”)) OR NOFT(”patient-robot” NEAR/2 (collaboration? or cooperation or interaction? OR ”shared space” OR ”sharing space”)) OR NOFT(”person-robot” NEAR/2 (collaboration? or cooperation or interaction? OR ”shared space” OR ”sharing space”)) OR NOFT(UR3 OR UR10 OR UR5 OR ”Universal Robot* 5” OR ”Universal Robot* 10” OR ”Universal Robot* 3” OR ”Franka Emika” OR ”KUKA LBR IIWA” OR ”Yumi robot”) OR NOFT(”Intelligent Assist Device” OR ”intelligent assist devices”) OR NOFT(“assist as needed”) OR NOFT(hybrid* NEAR/3 robot*) OR NOFT(ROBERT AND robot*)))

Set #2:
(NOFT((mca*) NEAR/3 (ischemi* or ischaemi* or infarct* or thrombo* or emboli* or occlus* or hypoxi* or haemorrhag* or hemorrhag* or haematoma* or hematoma* or bleed*)) OR NOFT((”anterior circulat*”) NEAR/3 (ischemi* or ischaemi* or infarct* or thrombo* or emboli* or occlus* or hypoxi* or haemorrhag* or hemorrhag* or haematoma* or hematoma* or bleed*))) AND (NOFT(rehab* OR telerehab* OR neurorehab* OR ”physical therap*” OR physiotherap* OR ”physio-therap*” OR ”occupational therap*”) OR NOFT(”motor relearn*” OR ”motor re-learn*” or ”motor learn*” or treatment or training or exercis*) OR NOFT(intervention NEAR/2 (robot* or mobilit*))) AND ((NOFT(co-bot? or cobot?) OR NOFT(cooperat* NEAR/3 robot*) OR NOFT(collab* NEAR/3 robot*) OR NOFT(”movement* primitive*”) OR NOFT(automat* NEAR/1 Robot*) OR NOFT(industrial* NEAR/1 Robot*) OR NOFT(reprogram* NEAR/1 Robot*) OR NOFT(re-program* NEAR/1 Robot*) OR NOFT(reconfig* NEAR/1 Robot*) OR NOFT(re-config* NEAR/1 Robot*) OR NOFT(”human-robot” NEAR/2 (collaboration? or cooperation or interaction? OR ”shared space” OR ”sharing space”)) OR NOFT(”patient-robot”

NEAR/2 (collaboration? or cooperation or interaction? OR ”shared space” OR ”sharing space”)) OR NOFT(”person-robot” NEAR/2 (collaboration? or cooperation or interaction? OR ”shared space” OR ”sharing space”)) OR NOFT(UR3 OR UR10 OR UR5 OR ”Universal Robot* 5” OR ”Universal Robot* 10” OR ”Universal Robot* 3” OR ”Franka Emika” OR ”KUKA LBR IIWA” OR ”Yumi robot”) OR NOFT(”Intelligent Assist Device” OR ”intelligent assist devices”) OR NOFT(“assist as needed”) OR NOFT(hybrid* NEAR/3 robot*) OR NOFT(ROBERT AND robot*)))

Set #3:
(NOFT((intracerebral) NEAR/3 (ischemi* or ischaemi* or infarct* or thrombo* or emboli* or occlus* or hypoxi* or haemorrhag* or hemorrhag* or haematoma* or hematoma* or bleed*)) OR NOFT((infratentorial) NEAR/3 (ischemi* or ischaemi* or infarct* or thrombo* or emboli* or occlus* or hypoxi* or haemorrhag* or hemorrhag* or haematoma* or hematoma* or bleed*)) OR NOFT((supratentorial) NEAR/3 (ischemi* or ischaemi* or infarct* or thrombo* or emboli* or occlus* or hypoxi* or haemorrhag* or hemorrhag* or haematoma* or hematoma* or bleed*))) AND (NOFT(rehab* OR telerehab* OR neurorehab* OR ”physical therap*” OR physiotherap* OR ”physio-therap*” OR ”occupational therap*”) OR NOFT(”motor relearn*” OR ”motor re-learn*” or ”motor learn*” or treatment or training or exercis*) OR NOFT(intervention NEAR/2 (robot* or mobilit*))) AND ((NOFT(co-bot? or cobot?) OR NOFT(cooperat* NEAR/3 robot*) OR NOFT(collab* NEAR/3 robot*) OR NOFT(”movement* primitive*”) OR NOFT(automat* NEAR/1 Robot*) OR NOFT(industrial* NEAR/1 Robot*) OR NOFT(reprogram* NEAR/1 Robot*) OR NOFT(re-program* NEAR/1 Robot*) OR NOFT(reconfig* NEAR/1 Robot*) OR NOFT(re-config* NEAR/1 Robot*) OR NOFT(”human-robot” NEAR/2 (collaboration? or cooperation or interaction? OR ”shared space” OR ”sharing space”)) OR NOFT(”patient-robot” NEAR/2 (collaboration? or cooperation or interaction? OR ”shared space” OR ”sharing space”)) OR NOFT(”person-robot” NEAR/2 (collaboration? or cooperation or interaction? OR ”shared space” OR ”sharing space”)) OR NOFT(UR3 OR UR10 OR UR5 OR ”Universal Robot* 5” OR ”Universal Robot* 10” OR ”Universal Robot* 3” OR ”Franka Emika” OR ”KUKA LBR IIWA” OR ”Yumi robot”) OR NOFT(”Intelligent Assist Device” OR ”intelligent assist devices”) OR NOFT(“assist as needed”) OR NOFT(hybrid* NEAR/3 robot*) OR NOFT(ROBERT AND robot*)))

Set #4:
(NOFT((vertebrobasil*) NEAR/3 (ischemi* or ischaemi* or infarct* or thrombo* or emboli* or occlus* or hypoxi* or haemorrhag* or hemorrhag* or haematoma* or hematoma* or bleed*)) OR NOFT((hemispher*) NEAR/3 (ischemi* or ischaemi* or infarct* or thrombo* or emboli* or occlus* or hypoxi* or haemorrhag* or hemorrhag* or haematoma* or hematoma* or bleed*)) OR NOFT((intracran*) NEAR/3 (ischemi* or ischaemi* or infarct* or thrombo* or emboli* or occlus* or hypoxi* or haemorrhag* or hemorrhag* or haematoma* or hematoma* or bleed*))) AND

(NOFT(rehab* OR telerehab* OR neurorehab* OR ”physical therap*” OR physiotherap* OR ”physio-therap*” OR ”occupational therap*”) OR NOFT(”motor relearn*” OR ”motor re-learn*” or ”motor learn*” or treatment or training or exercis*) OR NOFT(intervention NEAR/2 (robot* or mobilit*))) AND ((NOFT(co-bot? or cobot?) OR NOFT(cooperat* NEAR/3 robot*) OR NOFT(collab* NEAR/3 robot*) OR NOFT(”movement* primitive*”) OR NOFT(automat* NEAR/1 Robot*) OR NOFT(industrial* NEAR/1 Robot*) OR NOFT(reprogram* NEAR/1 Robot*) OR NOFT(re-program* NEAR/1 Robot*) OR NOFT(reconfig* NEAR/1 Robot*) OR NOFT(re-config* NEAR/1 Robot*) OR NOFT(”human-robot” NEAR/2 (collaboration? or cooperation or interaction? OR ”shared space” OR ”sharing space”)) OR NOFT(”patient-robot” NEAR/2 (collaboration? or cooperation or interaction? OR ”shared space” OR ”sharing space”)) OR NOFT(”person-robot” NEAR/2 (collaboration? or cooperation or interaction? OR ”shared space” OR ”sharing space”)) OR NOFT(UR3 OR UR10 OR UR5 OR ”Universal Robot* 5” OR ”Universal Robot* 10” OR ”Universal Robot* 3” OR ”Franka Emika” OR ”KUKA LBR IIWA” OR ”Yumi robot”) OR NOFT(”Intelligent Assist Device” OR ”intelligent assist devices”) OR NOFT(“assist as needed”) OR NOFT(hybrid* NEAR/3 robot*) OR NOFT(ROBERT AND robot*)))

Set #5:
(NOFT((brain) NEAR/3 (ischemi* or ischaemi* or infarct* or thrombo* or emboli* or occlus* or hypoxi* or haemorrhag* or hemorrhag* or haematoma* or hematoma* or bleed*)) OR NOFT((cerebr*) NEAR/3 (ischemi* or ischaemi* or infarct* or thrombo* or emboli* or occlus* or hypoxi* or haemorrhag* or hemorrhag* or haematoma* or hematoma* or bleed*)) OR NOFT((cerebell*) NEAR/3 (ischemi* or ischaemi* or infarct* or thrombo* or emboli* or occlus* or hypoxi* or haemorrhag* or hemorrhag* or haematoma* or hematoma* or bleed*))) AND (NOFT(rehab* OR telerehab* OR neurorehab* OR ”physical therap*” OR physiotherap* OR ”physio-therap*” OR ”occupational therap*”) OR NOFT(”motor relearn*” OR ”motor re-learn*” or ”motor learn*” or treatment or training or exercis*) OR NOFT(intervention NEAR/2 (robot* or mobilit*))) AND ((NOFT(co-bot? or cobot?) OR NOFT(cooperat* NEAR/3 robot*) OR NOFT(collab* NEAR/3 robot*) OR NOFT(”movement* primitive*”) OR NOFT(automat* NEAR/1 Robot*) OR NOFT(industrial* NEAR/1 Robot*) OR NOFT(reprogram* NEAR/1 Robot*) OR NOFT(re-program* NEAR/1 Robot*) OR NOFT(reconfig* NEAR/1 Robot*) OR NOFT(re-config* NEAR/1 Robot*) OR NOFT(”human-robot” NEAR/2 (collaboration? or cooperation or interaction? OR ”shared space” OR ”sharing space”)) OR NOFT(”patient-robot” NEAR/2 (collaboration? or cooperation or interaction? OR ”shared space” OR ”sharing space”)) OR NOFT(”person-robot” NEAR/2 (collaboration? or cooperation or interaction? OR ”shared space” OR ”sharing space”)) OR NOFT(UR3 OR UR10 OR UR5 OR ”Universal Robot* 5” OR ”Universal Robot* 10” OR ”Universal Robot* 3” OR ”Franka Emika” OR ”KUKA LBR IIWA” OR ”Yumi robot”) OR NOFT(”Intelligent Assist Device” OR ”intelligent assist devices”) OR NOFT(“assist as needed”) OR NOFT(hybrid* NEAR/3 robot*) OR NOFT(ROBERT AND robot*)))

Set #6: ((NOFT(stroke OR strokes OR poststroke OR poststrokes OR hemipleg* OR hemipar* OR paresis OR paretic OR apoplex* OR "cerebral vasc*" OR cerebrovasc* OR cva OR tia OR "transient ischemic attack" OR "transient ischaemic attack" OR SCI OR spine OR spinal OR parapleg* OR tetrapleg* OR quadripleg* OR "upper limb*" OR "upper extremit*" OR "lower limb*" OR "lower extremit*" OR arm OR arms OR shoulder OR shoulders OR hand OR hands OR axilla OR elbow OR elbows OR forearm OR forearms OR finger OR fingers OR wrist OR wrists OR dexterity OR dextrous OR grip OR gripping OR grasp OR grasping OR tap OR tapping OR reach OR reaching OR leg OR legs OR foot OR feet OR thigh* OR knee* OR ankle* OR balance OR gait* OR walk* OR ambulat* OR stabilit* OR mobilit* OR locomot* OR step* OR stride OR strides)) AND (NOFT(rehab* OR telerehab* OR neurorehab* OR "physical therap*" OR physiotherap* OR "physio-therap*" OR "occupational therap*") OR NOFT("motor relearn*" OR "motor re-learn*" OR "motor learn*" OR treatment OR training OR exercis*) OR NOFT(intervention NEAR/2 (robot* OR mobilit*))) AND ((NOFT(co-bot? OR cobot?) OR NOFT(cooperat* NEAR/3 robot*) OR NOFT(collab* NEAR/3 robot*) OR NOFT("movement* primitive*") OR NOFT(automat* NEAR/1 Robot*) OR NOFT(industrial* NEAR/1 Robot*) OR NOFT(reprogram* NEAR/1 Robot*) OR NOFT(re-program* NEAR/1 Robot*) OR NOFT(reconfig* NEAR/1 Robot*) OR NOFT(re-config* NEAR/1 Robot*) OR NOFT("human-robot" NEAR/2 (collaboration? OR cooperation OR interaction? OR "shared space" OR "sharing space")) OR NOFT("patient-robot" NEAR/2 (collaboration? OR cooperation OR interaction? OR "shared space" OR "sharing space")) OR NOFT("person-robot" NEAR/2 (collaboration? OR cooperation OR interaction? OR "shared space" OR "sharing space")) OR NOFT(UR3 OR UR10 OR UR5 OR "Universal Robot* 5" OR "Universal Robot* 10" OR "Universal Robot* 3" OR "Franka Emika" OR "KUKA LBR IIWA" OR "Yumi robot") OR NOFT("Intelligent Assist Device" OR "intelligent assist devices") OR NOFT(“assist as needed”) OR NOFT(hybrid* NEAR/3 robot*) OR NOFT(ROBERT AND robot*)))) AND la.exact("ENG")

Database: COMPENDEX
Platform: Engineering village
Date last searched November 28, 2024

(((((((stroke OR strokes OR poststroke OR poststrokes OR hemipleg* OR hemipar* OR paresis OR paretic OR apoplex* OR cerebral vasc* OR cerebrovasc* OR cva OR tia OR transient ischemic attack OR transient ischaemic attack OR SCI OR spine OR spinal OR parapleg* OR tetrapleg* OR quadripleg* OR upper limb* OR upper extremit* OR lower limb* OR lower extremit* OR arm OR arms OR shoulder OR shoulders OR hand OR hands OR axilla OR elbow OR elbows OR forearm OR forearms OR finger OR fingers OR wrist OR wrists OR dexterity OR dextrous OR grip OR gripping OR grasp OR grasping OR tap OR tapping OR reach OR reaching OR leg OR legs OR foot OR feet OR thigh* OR knee* OR ankle* OR balance OR gait* OR walk* OR ambulat* OR stabilit* OR mobilit* OR locomot* OR step* OR stride OR strides) WN KY)) AND (1884-2024 WN YR)) AND ((((((rehab* OR telerehab* OR neurorehab* OR physical therap* OR physiotherap* OR physio therap* OR

occupational therap*) WN ALL) OR ((motor relearn* OR motor re-learn* OR motor learn* OR treatment OR training OR exercis*) WN KY) OR ((intervention NEAR/2 $robot) WN KY) OR ((intervention NEAR/2 $mobility) WN KY)) AND (1666-2024 WN YR)) AND ((((co-bot* OR cobot*) WN KY) OR ((cooperative NEAR/3 $robot) WN KY) OR ((collaborative NEAR/3 $robot) WN KY) OR ((movement primitive) WN KY) OR ((automated NEAR/1 $robot) WN KY) OR ((industrial NEAR/1 $robot) WN KY) OR ((reprogram NEAR/1 $robot) WN KY) OR ((reprogramed NEAR/1 $robot) WN KY) OR ((re-program NEAR/1 $robot) WN KY) OR ((re-programmed NEAR/1 $robot) WN KY) OR ((reconfigured NEAR/1 $robot) WN KY) OR ((re-configured NEAR/1 $robot) WN KY) OR ((human-robot NEAR/2 collaboration) WN KY) OR ((human-robot NEAR/2 cooperation) WN KY) OR ((human-robot NEAR/2 $interaction) WN KY) OR ((human-robot NEAR/2 shared space) WN KY) OR ((human-robot NEAR/2 sharing space) WN KY) OR ((patient-robot NEAR/2 collaboration) WN KY) OR ((patient-robot NEAR/2 cooperation) WN KY) OR ((patient-robot NEAR/2 $interaction) WN KY) OR ((patient-robot NEAR/2 shared space) WN KY) OR ((patient-robot NEAR/2 sharing space) WN KY) OR ((person-robot NEAR/2 collaboration) WN KY) OR ((person-robot NEAR/2 $cooperation) WN KY) OR ((person-robot NEAR/2 $interaction) WN KY) OR ((person-robot NEAR/2 shared space) WN KY) OR ((person-robot NEAR/2 sharing space) WN KY) OR ((UR3 OR UR10 OR UR5 OR Universal Robot 5 OR Universal Robot 10 OR Universal Robot 3 OR Franka Emika OR KUKA LBR IIWA OR Yumi robot) WN KY) OR ((Intelligent Assist Device OR intelligent assist devices) WN KY) OR ((“assist as needed”) WN KY) OR (($hybrid NEAR/3 $robot) WN KY) OR ((ROBERT AND $robot) WN KY)) AND (1666-2024 WN YR))) AND (1884-2024 WN YR)))) AND (english WN LA))

Database: INSPEC
Platform: Engineering Village
Date last searched November 28, 2024
(((((((stroke OR strokes OR poststroke OR poststrokes OR hemipleg* OR hemipar* OR paresis OR paretic OR apoplex* OR cerebral vasc* OR cerebrovasc* OR cva OR tia OR transient ischemic attack OR transient ischaemic attack OR SCI OR spine OR spinal OR parapleg* OR tetrapleg* OR quadripleg* OR upper limb* OR upper extremit* OR lower limb* OR lower extremit* OR arm OR arms OR shoulder OR shoulders OR hand OR hands OR axilla OR elbow OR elbows OR forearm OR forearms OR finger OR fingers OR wrist OR wrists OR dexterity OR dextrous OR grip OR gripping OR grasp OR grasping OR tap OR tapping OR reach OR reaching OR leg OR legs OR foot OR feet OR thigh* OR knee* OR ankle* OR balance OR gait* OR walk* OR ambulat* OR stabilit* OR mobilit* OR locomot* OR step* OR stride OR strides) WN KY)) AND (1884-2024 WN YR)) AND ((((((rehab* OR telerehab* OR neurorehab* OR physical therap* OR physiotherap* OR physio therap* OR occupational therap*) WN ALL) OR ((motor relearn* OR motor re-learn* OR motor learn* OR treatment OR training OR exercis*) WN KY) OR ((intervention NEAR/2 $robot) WN KY) OR ((intervention NEAR/2 $mobility) WN KY)) AND (1666-2024 WN YR)) AND ((((co-bot* OR cobot*) WN KY) OR ((cooperative NEAR/3 $robot)

WN KY) OR ((collaborative NEAR/3 $robot) WN KY) OR ((movement primitive) WN KY) OR ((automated NEAR/1 $robot) WN KY) OR ((industrial NEAR/1 $robot) WN KY) OR ((reprogram NEAR/1 $robot) WN KY) OR ((reprogramed NEAR/1 $robot) WN KY) OR ((re-program NEAR/1 $robot) WN KY) OR ((re-programmed NEAR/1 $robot) WN KY) OR ((reconfigured NEAR/1 $robot) WN KY) OR ((re-configured NEAR/1 $robot) WN KY) OR ((human-robot NEAR/2 collaboration) WN KY) OR ((human-robot NEAR/2 cooperation) WN KY) OR ((human-robot NEAR/2 $interaction) WN KY) OR ((human-robot NEAR/2 shared space) WN KY) OR ((human-robot NEAR/2 sharing space) WN KY) OR ((patient-robot NEAR/2 collaboration) WN KY) OR ((patient-robot NEAR/2 cooperation) WN KY) OR ((patient-robot NEAR/2 $interaction) WN KY) OR ((patient-robot NEAR/2 shared space) WN KY) OR ((patient-robot NEAR/2 sharing space) WN KY) OR ((person-robot NEAR/2 collaboration) WN KY) OR ((person-robot NEAR/2 $cooperation) WN KY) OR ((person-robot NEAR/2 $interaction) WN KY) OR ((person-robot NEAR/2 shared space) WN KY) OR ((person-robot NEAR/2 sharing space) WN KY) OR ((UR3 OR UR10 OR UR5 OR Universal Robot 5 OR Universal Robot 10 OR Universal Robot 3 OR Franka Emika OR KUKA LBR IIWA OR Yumi robot) WN KY) OR ((Intelligent Assist Device OR intelligent assist devices) WN KY) OR ((“assist as needed”) WN KY) OR (($hybrid NEAR/3 $robot) WN KY) OR ((ROBERT AND $robot) WN KY)) AND (1666-2024 WN YR))) AND (1884-2024 WN YR)))) AND (english WN LA))

Database: ACM Digital Library
Platform: N/A
Date last searched November 28, 2024
(Title:(”co-bot” OR ”co-bots” OR cobot OR ”collaborative robot” OR ”collaborative robots” OR ”automated robot” OR ”automated robots” OR ”industrial robot” OR ”industrial robots” OR ur3 OR ur10 OR ur5 OR ”universal robot 5” OR ”universal robots 5” OR ”universal robot 10” OR ”universal robots 10” OR ”universal robot 3” OR ”universal robots 3” OR ”franka emika” OR ”kuka lbr iiwa” OR ”yumi robot” OR ”intelligent assist device” OR ”intelligent assist devices” OR ”human-robot” OR ”patient-robot” ”person-robot” OR ”assist as needed” OR ”hybrid robot” OR ”hybrid robots” OR ”hybrid robotic” OR ”hybrid robotics” OR (ROBERT robot*)) OR Abstract:(”co-bot” OR ”co-bots” OR cobot OR ”collaborative robot” OR ”collaborative robots” OR ”automated robot” OR ”automated robots” OR ”industrial robot” OR ”industrial robots” OR ur3 OR ur10 OR ur5 OR ”universal robot 5” OR ”universal robots 5” OR ”universal robot 10” OR ”universal robots 10” OR ”universal robot 3” OR ”universal robots 3” OR ”franka emika” OR ”kuka lbr iiwa” OR ”yumi robot” OR ”intelligent assist device” OR ”intelligent assist devices” OR ”human-robot” OR ”patient-robot” OR ”person-robot” OR ”assist as needed” OR ”hybrid robot” OR ”hybrid robots” OR ”hybrid robotic” OR ”hybrid robotics” OR (ROBERT robot*))) AND (Title:(rehab* OR telerehab* OR neurorehab* OR ”occupational therapy” OR ”occupational therapist” OR ”physical therapy” OR ”physical therapist” OR ”physio-therapy” OR ”physio-therapist” OR physiotherap* OR ”motor learning” OR ”motor relearning” OR ”motor re-learning” OR ”mobility

intervention") OR Abstract:(rehab* OR telerehab* OR neurorehab* OR "occupational therapy" OR "occupational therapist" OR "physical therapy" OR "physical therapist" OR "physio-therapy" OR "physio-therapist" OR physiotherap* OR "motor learning" OR "motor relearning" OR "motor re-learning" OR "mobility intervention")) AND (Title:(stroke OR strokes OR poststroke OR hemipleg* OR hemipar* OR paresis OR paretic OR apoplex* OR cerebrovascular OR "cerebal vascular" OR "transient ischemic attack" OR "transient ischaemic attack" OR SCI OR spine OR spinal OR parapleg* OR tetrapleg* OR quadripleg* OR "upper limb" OR "upper limbs" OR "upper extremity" OR "upper extremities" OR "lower limb" OR "lower limbs" OR "lower extremity" OR "lower extremities" OR arm OR arms OR shoulder OR shoulders OR hand OR hands OR axilla OR elbow OR elbows OR forearm OR forearms OR finger OR fingers OR wrist OR wrists OR dexterity OR dextrous OR grip OR gripping OR grasp OR grasping OR tap tapping OR reach OR reaching OR leg OR legs OR foot OR feet OR thigh* OR knee* OR ankle* OR balance OR gait* OR walk* OR ambulat* OR stabilit* OR mobilit* OR locomot* OR step* OR stride OR strides) OR Abstract:(stroke OR strokes OR poststroke OR hemipleg* OR hemipar* OR paresis OR paretic OR apoplex* OR cerebrovascular OR "cerebal vascular" OR "transient ischemic attack" OR "transient ischaemic attack" OR SCI OR spine OR spinal OR parapleg* OR tetrapleg* OR quadripleg* OR "upper limb" OR "upper limbs" OR "upper extremity" OR "upper extremities" OR "lower limb" OR "lower limbs" OR "lower extremity" OR "lower extremities" OR arm OR arms OR shoulder OR shoulders OR hand OR hands OR axilla OR elbow OR elbows OR forearm OR forearms OR finger OR fingers OR wrist OR wrists OR dexterity OR dextrous OR grip OR gripping OR grasp OR grasping OR tap tapping OR reach OR reaching OR leg OR legs OR foot OR feet OR thigh* OR knee* OR ankle* OR balance OR gait* OR walk* OR ambulat* OR stabilit* OR mobilit* OR locomot* OR step* OR stride OR strides))

Database: IEEE Xplore
Platform: N/A
Date last searched November 28, 2024
("All Metadata":"co-bot" OR "All Metadata":"co-bots" OR "All Metadata":cobot OR "All Metadata":"collaborative robot" OR "All Metadata":"collaborative robots" OR "All Metadata":"automated robot" OR "All Metadata":"automated robots" OR "All Metadata":"industrial robot" OR "All Metadata":"industrial robots" OR "All Metadata":ur3 OR "All Metadata":ur10 OR "All Metadata":ur5 OR "All Metadata":"universal robot 5" OR "All Metadata":"universal robots 5" OR "All Metadata":"universal robot 10" OR "All Metadata":"universal robots 10" OR "All Metadata":"universal robot 3" OR "All Metadata":"universal robots 3" OR "All Metadata":"franka emika" OR "All Metadata":"kuka lbr iiwa" OR "All Metadata":"yumi robot" OR "All Metadata":"intelligent assist device" OR "All Metadata":"intelligent assist devices" OR "All Metadata":"human-robot" OR "All Metadata":"patient-robot" OR "All Metadata":"person-robot" OR "All Metadata":"assist as needed" OR "All Metadata":"hybrid robot" OR "All Metadata":"hybrid robots" OR "All Metadata":"hybrid robotic" OR "All

Metadata":"hybrid robotics" OR "All Metadata":ROBERT robot*) AND ("All Metadata":rehab OR "All Metadata":rehabilitation OR "All Metadata":telerehab OR "All Metadata":telerehabilitation OR "All Metadata":neurorehab OR "All Metadata":neurorehabilitation OR "All Metadata":"occupational therapy" OR "All Metadata":"occupational therapist" OR "All Metadata":"physical therapy" OR "All Metadata":"physical therapist" OR "All Metadata":"physio-therapy" OR "All Metadata":"physio-therapist" OR "All Metadata":physiotherapy OR "All Metadata":physiotherapist OR "All Metadata":"motor learning" OR "All Metadata":"motor relearning" OR "All Metadata":"motor re-learning" OR "All Metadata":"mobility intervention") AND ("All Metadata":stroke OR "All Metadata":strokes OR "All Metadata":poststroke OR "All Metadata":hemipleg* OR "All Metadata":hemipar* OR "All Metadata":paresis OR "All Metadata":paretic OR "All Metadata":apoplex* OR "All Metadata":cerebrovascular OR "All Metadata":"cerebal vascular" OR "All Metadata":"transient ischemic attack" OR "All Metadata":"transient ischaemic attack" OR "All Metadata":SCI OR "All Metadata":spine OR "All Metadata":spinal OR "All Metadata":parapleg* OR "All Metadata":tetrapleg* OR "All Metadata":quadripleg* OR "All Metadata":"upper limb" OR "All Metadata":"upper limbs" OR "All Metadata":"upper extremity" OR "All Metadata":"upper extremities" OR "All Metadata":"lower limb" OR "All Metadata":"lower limbs" OR "All Metadata":"lower extremity" OR "All Metadata":"lower extremities" OR "All Metadata":arm OR "All Metadata":arms OR "All Metadata":shoulder OR "All Metadata":shoulders OR "All Metadata":hand OR "All Metadata":hands OR "All Metadata":axilla OR "All Metadata":elbow OR "All Metadata":elbows OR "All Metadata":forearm OR "All Metadata":forearms OR "All Metadata":finger OR "All Metadata":fingers OR "All Metadata":wrist OR "All Metadata":wrists OR "All Metadata":dexterity OR "All Metadata":dextrous OR "All Metadata":grip OR "All Metadata":gripping OR "All Metadata":grasp OR "All Metadata":grasping OR "All Metadata":tap OR "All Metadata":tapping OR "All Metadata":reach OR "All Metadata":reaching OR "All Metadata": leg OR "All Metadata":legs OR "All Metadata":foot OR "All Metadata":feet OR "All Metadata":thigh OR "All Metadata":thighs OR "All Metadata":knee OR "All Metadata":knees OR "All Metadata":ankle OR "All Metadata":ankles OR "All Metadata":balance OR "All Metadata":gait OR "All Metadata":walk OR "All Metadata":walking OR "All Metadata":walks OR "All Metadata":walked OR "All Metadata":ambulate OR "All Metadata":ambulation OR "All Metadata":stability OR "All Metadata":stabilize OR "All Metadata":mobility OR "All Metadata":locomotion OR "All Metadata":locomote OR "All Metadata":step* OR "All Metadata":stride OR "All Metadata":strides)
